# Graphene-based RRAM devices for neural computing

**DOI:** 10.3389/fnins.2023.1253075

**Published:** 2023-10-05

**Authors:** Rajalekshmi T. R, Rinku Rani Das, Chithra Reghuvaran, Alex James

**Affiliations:** Digital University, Thiruvananthapuram, Kerala, India

**Keywords:** chemical vapor deposition (CVD), cryptography, graphene, neuromorphic computing, resistive random access memory (RRAM)

## Abstract

Resistive random access memory is very well known for its potential application in in-memory and neural computing. However, they often have different types of device-to-device and cycle-to-cycle variability. This makes it harder to build highly accurate crossbar arrays. Traditional RRAM designs make use of various filament-based oxide materials for creating a channel that is sandwiched between two electrodes to form a two-terminal structure. They are often subjected to mechanical and electrical stress over repeated read-and-write cycles. The behavior of these devices often varies in practice across wafer arrays over these stresses when fabricated. The use of emerging 2D materials is explored to improve electrical endurance, long retention time, high switching speed, and fewer power losses. This study provides an in-depth exploration of neuro-memristive computing and its potential applications, focusing specifically on the utilization of graphene and 2D materials in RRAM for neural computing. The study presents a comprehensive analysis of the structural and design aspects of graphene-based RRAM, along with a thorough examination of commercially available RRAM models and their fabrication techniques. Furthermore, the study investigates the diverse range of applications that can benefit from graphene-based RRAM devices.

## 1. Introduction

Graphene-based resistive random access memory (RRAM) devices have gained significant attention in recent years for their potential applications in neural computing. Graphene, a two-dimensional carbon material, has exceptional electrical and mechanical properties, making it an attractive candidate for RRAM devices. RRAM is considered one of the most promising emerging non-volatile memory, a potentially universal memory device that comes under the broad category of memristive systems (Meena et al., 2014). The advantage of RRAM is attributed to the ease of fabrication of a two-terminal structure that can be used to create efficient crossbar arrays, high read speeds, and low area overheads. The RRAMs in the crossbar can emulate multiply and accumulate (MAC) computations that are universal operations essential for implementing neural computations.

RRAM is a memory based on a resistive switching mechanism where the conducting filament is created and broken due to a change of external voltage (Yu et al., 2011a). The binary RRAMs operate in two states: low resistance state (LRS) and high resistance state (HRS). Various types of electrodes and metal oxides can be used for RRAM structure. Titanium, hafnium, silicon, germanium, and nickel are the most common oxide materials, whereas silicon, silver, indium, and tantalum are familiar electrode materials used in RRAM memory devices.

Unfortunately, RRAM memory devices face various limitations with the aforementioned electrode and oxide materials (Zhu et al., 2015). For accomplishing the resistive switching property, the electrode, and conducting filament can be modified with a wide variety of materials. The electrode materials used for RRAM are divided into the following five categories: (i) elementary substance electrodes, (ii) silicon-based electrodes, (iii) alloy electrodes, (iv) oxide electrodes, and (v) nitride-based electrodes (Zahoor et al., 2020). Depending on the electrode material, the number of possible states in the RRAM varies (Prakash and Hwang, 2019). As the number of states increases, the device finds application as an analog data storage device.

In RRAM, the graphene-related materials have been incorporated to increase the switching speed, retention time, endurance, and power consumption to improve the performance as a non-volatile memory (Rehman et al., 2020). Graphene provides additional properties such as transparency, flexibility, enhanced heat dissipation due to the high thermal conductivity of graphene, and chemical stability. Other than these properties, as a two-dimensional system, graphene can provide more than two states for the memristive device in implementing synapses for neuromorphic computing. It is reported that till now more than 16 states are possible with graphene in the memristive system (Schranghamer et al., 2020). Building more than two stable states in RRAMs to form analog computing systems or using them for analog storage is a open problem in RRAM-based systems.

With graphene-enabled RRAMs, it is expected that the higher number of states can improve the storage density and improve the reliability of the device. Graphene-enhanced RRAM exhibits faster switching speeds and enduring performance due to high carrier mobility, and the unique two-dimensional structure minimizes filament variability, ensuring stable set/reset processes in RRAM devices. Exceptional thermal and mechanical stability of graphene boosts RRAM features by optimizing performance across varying conditions (Galashev and Rakhmanova, 2014; Pan et al., 2017; Rehman et al., 2020). It is reported that RRAM devices offer a switching speed of less than 10 ns, power losses of about 10 pJ, lower threshold voltage of less than 1V, long retention time of greater than 10 years, high electrical endurance with more than 10^8^ voltage cycles, and extended mechanical robustness of 500 bending cycles. These advantages are complemented by its ability to tolerate high-temperature variations. Graphene as an interface layer acts as a resistive switching medium which help to minimize power dissipation with low contact resistance. Graphene helps to optimize the surface effect such as photodesorption and chemisorption which are varied due to the increase and decrease of the temperature.

This review starts with an overview of neuro-memristive computing, graphene, and its synthesis techniques. Furthermore, the RRAM, working principle, and the resistive switching mechanism are discussed. The incorporation of graphene and graphene oxide in RRAM as an electrode, and the middle layer is elaborated in detail. The role of graphene in RRAM, to enhance the properties such as endurance, and retention is analyzed, and the enhancement in flexibility and transparency is discussed. The progress of multilevel cell storage in RRAM is reviewed in detail. Furthermore, the commercially available RRAM models and their fabrication methods, complementary metal-oxide-semiconductor (CMOS) compatibility with RRAM are also discussed.

## 2. Neuro-memristive computing

### 2.1. Memristive devices and neural dynamics

Memristive devices have been studied for their potential to create artificial neural networks that can learn and adapt in a manner similar to biological neural networks (Huang et al., 2020). These devices can be used to build artificial synapses that can modify their strength based on the pattern of electrical signals they receive. This is similar to how biological synapses modify their strength in response to the timing and frequency of incoming electrical signals (Zhang et al., 2023). Based on this, one potential application of memristive devices in neural dynamics is in the development of neuromorphic computing systems (Ma et al., 2018). These systems are designed to mimic the way the brain processes information, and memristive devices could provide a way to build artificial neural networks that are more efficient and flexible than traditional computing systems (Shehab et al., 2022). This section will cover the details of different kinds of memristive devices, their working, and their viability for application in neuromorphic computing systems.

**Figure 1:**
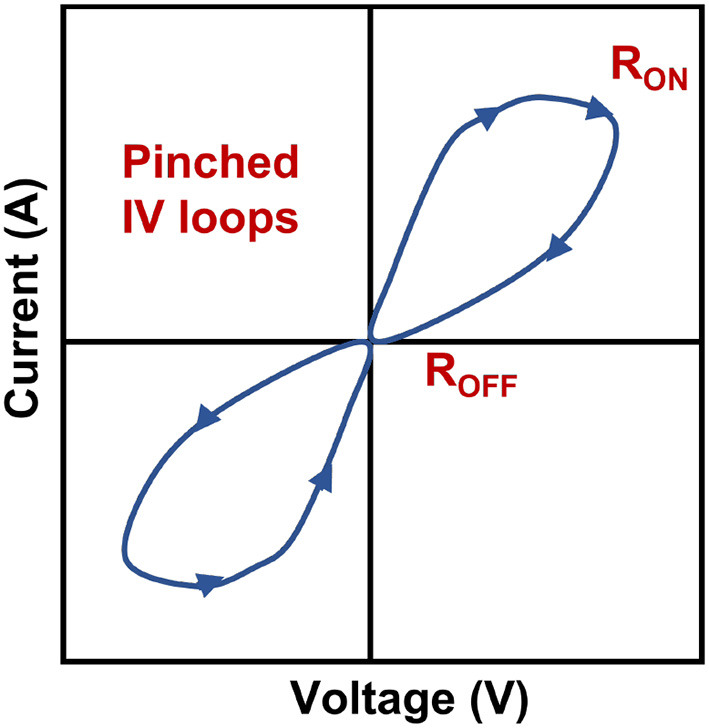
Example of pinched hysteresis loop of memristor.

**Figure 2:**
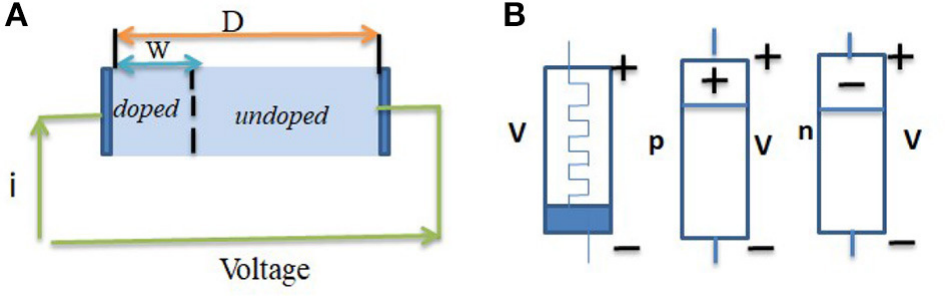
**(A)** Memristor model according to Strukov et al. (2008). **(B)** Traditional symbol, p-type and n-type memristors (copyright Starzyk et al., 2014).

Memristor is one kind of two-terminal device, considered a new-generation non-volatile memory (NVM) device. This new computing system proposed by Sano et al. (2013) can store information by changing the resistance of a material, whereas conventional memory devices program data by change of capacitance (Im et al., 2020). A pinched hysteresis loop is a characteristic feature of a memristor. The loop represents the behavior of the memristor as the voltage or current applied to it is varied as shown in Figure 1. The pinched hysteresis loop is a distinctive characteristic of memristors and distinguishes them from other electronic devices such as resistors, capacitors, and inductors. The pinched hysteresis loop arises due to the inherent properties of the memristor's material and structure, which allow it to exhibit memory and resistance variations based on the history of applied voltage or current. The exact shape and characteristics of the loop depend on the specific properties of the memristor, including its materials, fabrication methods, and operating conditions. The pinched hysteresis loop of a memristor has significant implications for applications in areas such as memory devices, neuromorphic computing, and analog signal processing. It enables the memristor to store information based on its resistance state and offers unique opportunities for non-volatile memory and computing architectures. The conventional memristor model and its symbol are shown in Figures 2A, B.

**Figure 3:**
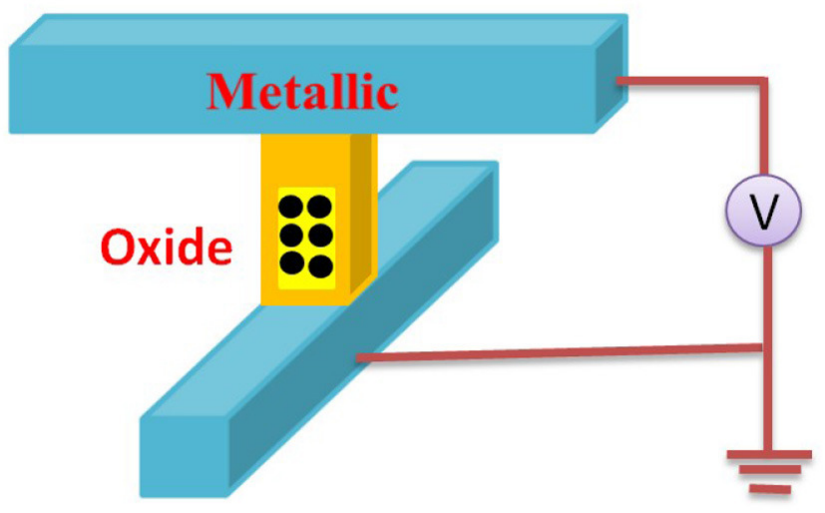
Schematic of the cross-point device, showing metallic top and bottom electrodes, and switching oxide (Yang and Williams, 2013).

These devices offer several advantages over conventional memory technologies such as flash, dynamic random access memory (DRAM), and static random access memory (SRAM), including high density, low power consumption, and fast switching speeds (Yang and Williams, 2013). The combination of metal electrodes and insulators constructs a memristor configuration. The schematic diagram of the cross-point device, showing metallic top and bottom electrodes and switching oxide is shown in Figure 3. Resistive switching, phase change, spintronics ferroelectric, etc. are the various kinds of properties of memristor devices that are contributing to the development of emerging electronic technologies. Among them, a resistive switching memristor (RSM) is the most common memristive device which has low power consumption, high endurance, and potential for use in neuromorphic computing (Prodromakis and Toumazou, 2010; Yu et al., 2018). The applied voltage to the electrodes in the RSM device creates an electric field across the metal oxide layer, causing a change in the oxidation state of the material. This oxidation state changes the resistance of the material which can be detected and used to store data. Phase change element based phase change memory (PCM) is another type of memristive device that uses a material to change its physical state between a crystalline phase (low resistance) and an amorphous phase (high resistance) in response to heat or electric current. Spintronics memristors are a new type of magnetic RAM (MRAM) that works on magnetic tunnel junction (MTJ) (Xue et al., 2011) and offers high speed and high endurance performance. The resistance value has changed due to the spin of the electron and the storage of the data. Two ferromagnetic layers (FM) of these devices are separated by a non-magnetic (NM) layer. When an electric current is applied to the device, the spin of electrons in the magnetic layers is affected, causing a change in the resistance of the device. Ferroelectric tunnel junction (FTJ) (Ambriz-Vargas et al., 2017) is the most significant ferroelectric memory device for neuromorphic computation, having an insulating layer in between two metal electrodes. This ferroic nanostructure is comprised of an ultra-thin ferroelectric barrier, and its dominant mechanism is quantum electron tunneling. In this structure, electrons are able to penetrate through the potential barrier of the ultra-thin insulator. As research in this field continues to progress, memristive devices are expected to play an increasingly important role in the development of advanced computing and memory technologies.

Memristive devices are of great interest in the field of neuromorphic computing because they can be used to emulate the synaptic connections between neurons in the brain. The neural dynamics of memristive devices refers to the behavior of these devices when they are used to implement neural networks. When memristive devices are used as synapses in a neural network, their resistance values change over time in response to the input signals that they receive (Boybat et al., 2018). This behavior can be used to implement learning in the neural network, allowing it to adapt to new inputs and improve its performance over time. The dynamics of memristive devices in neural networks are highly non-linear and can be difficult to predict (Brivio et al., 2021). However, researchers have developed models and simulations to study the behavior of these devices in neural networks.

### 2.2. Memristors in crossbar

Memristors in crossbar arrays are a type of non-volatile memory technology that holds promise for high-density, low-power, and high-speed computing applications (Xia and Yang, 2019). In a crossbar array, memristors are arranged in a grid pattern, with one set of wires running vertically and another set of wires running horizontally, forming a series of intersecting points. At each cross-point, a memristor can be programmed to either a high or low resistance state, representing a binary 1 or 0, respectively. By applying voltage to the appropriate sets of wires, the resistance state of the memristor can be read or written. This allows for parallel access to multiple memory cells, making crossbar arrays a potential solution for memory-intensive tasks such as machine learning and artificial intelligence.

A single memristor or one-transistor/one-resistor (1T, 1R) memristor array typically refers to a configuration where memristors are organized in a regular grid pattern. The purpose of a single memristor array is to enable the simultaneous operation and interconnection of multiple memristors (Xu et al., 2021). In a 1T, 1R memristor array, each memristor is paired with a transistor. The transistor serves as the access device or switch, allowing individual memristors within the array to be addressed and read or written to Kim et al. (2012). The key advantage of a 1T, 1R memristor array is its high density and potential for low-power operation. By combining the storage element (memristor) and the access device (transistor) into a single unit, the overall footprint of the memory array can be reduced. There are various ways to arrange the memristors, depending on the desired application and circuit design (Lu et al., 2022). The two-memristor crossbar array is a grid-like structure where the two memristors are positioned at the intersection of a row and a column. The rows and columns are connected to input and output nodes or other circuit elements. This configuration is commonly used in memristive crossbar arrays, where the resistance states of the memristors can be manipulated to enable or disable the connections between rows and columns (Vourkas et al., 2016). Crossbar arrays are particularly relevant in applications such as memory arrays, neural networks, and digital logic circuits (Li et al., 2021). In a bridge memristive crossbar array, two memristors are connected in series between two nodes, forming a bridge structure. The nodes can represent inputs, outputs, or intermediate connections in a larger circuit. The bridge configuration allows for specific control over the flow of current or signals through the array. By adjusting the resistance states of the individual memristors in the bridge, it is possible to selectively enable or disable the connection between the two nodes. This can be achieved by applying appropriate voltage or current across the bridge.

Memristors in crossbar arrays also have the potential for use in neuromorphic computing, which seeks to emulate the structure and function of the human brain (Xia and Yang, 2019). Memristor-based crossbar arrays can potentially perform tasks such as pattern recognition and decision-making in a highly efficient and parallelized manner. Starzyk et al. (2014) developed a novel neural network architecture that utilizes a compact crossbar layout of memristors, which allows us to preserve a high density of synaptic connections. Yakopcic et al. (2019) studied a memristor-based neuromorphic system for ex-situ training of multi-layer perceptron algorithms. This technique facilitates the direct translation of neural algorithm weights onto the resistive grid of a memristor crossbar. It is observed that a parallel crossbar improves the speed and power dissipation. Hu et al. (2012b) proposed a memristive crossbar array for high-speed image processing. It exhibits automatic memory, continuous output, and high-speed parallel computation, making it well suited for implementation in VLSI (very large-scale integration) technology. Huang et al. (2021) developed a vertical crossbar MIM (metal insulator metal) RRAM device for neuromorphic computing that is based on the 2D material ReSe_2_. This design has been shown to exhibit improved accuracy when used in brain-inspired neuromorphic computing systems.

### 2.3. Neuro-memristive architectures

The memristive circuits and computing architectures are one of the promising solutions for implementing neuromorphic computing. The memristor implementations provide various advantages such as scalability, on-chip area and power reduction, efficiency, and adaptability, especially for device scale-up architectures. There are existing different memristive neuromorphic architectures in the literature used for edge computing applications. The section reviews the most popular neural architectures for edge computing applications.

#### 2.3.1. Deep neural network (DNN)

**Figure 4:**
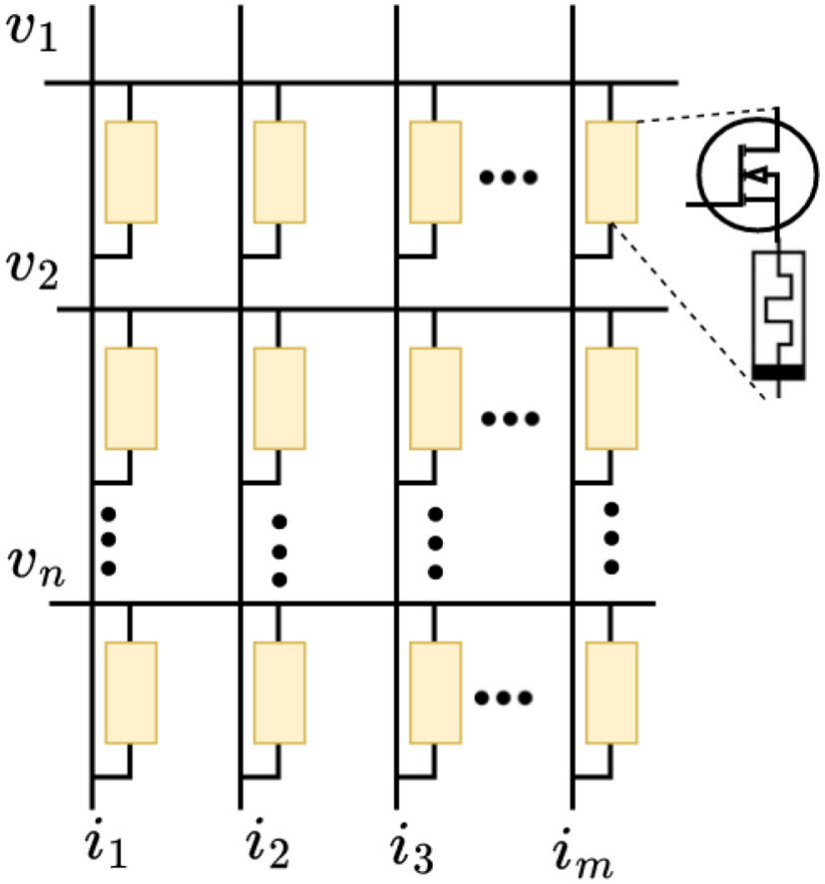
ITIM configuration for implementing DNN neural network.

The DNN is implemented using memristor crossbar arrays. Each DNN layer is implemented using one transistor/memristor (1T 1M) configuration as in Figure 4. Each layer consists of *M* word lines (WLs) and *N* bit lines (BLs). The transistor switch enables or disables the column-wise memristor nodes. In Figure 4, *v*_1_, *v*_2_, … *v*_*n*_ from the inputs, conductance *g*_*i, j*_ of memristors as weights and columns current *i*_1_, *i*_2_, … *i*_*m*_ as outputs, where *i, j* are the coordinates of the crossbar node. The output currents indicate the weighted summation of input voltages. The bias is included as an additional input line.

#### 2.3.2. Convolutional neural network (CNN)

**Figure 5:**
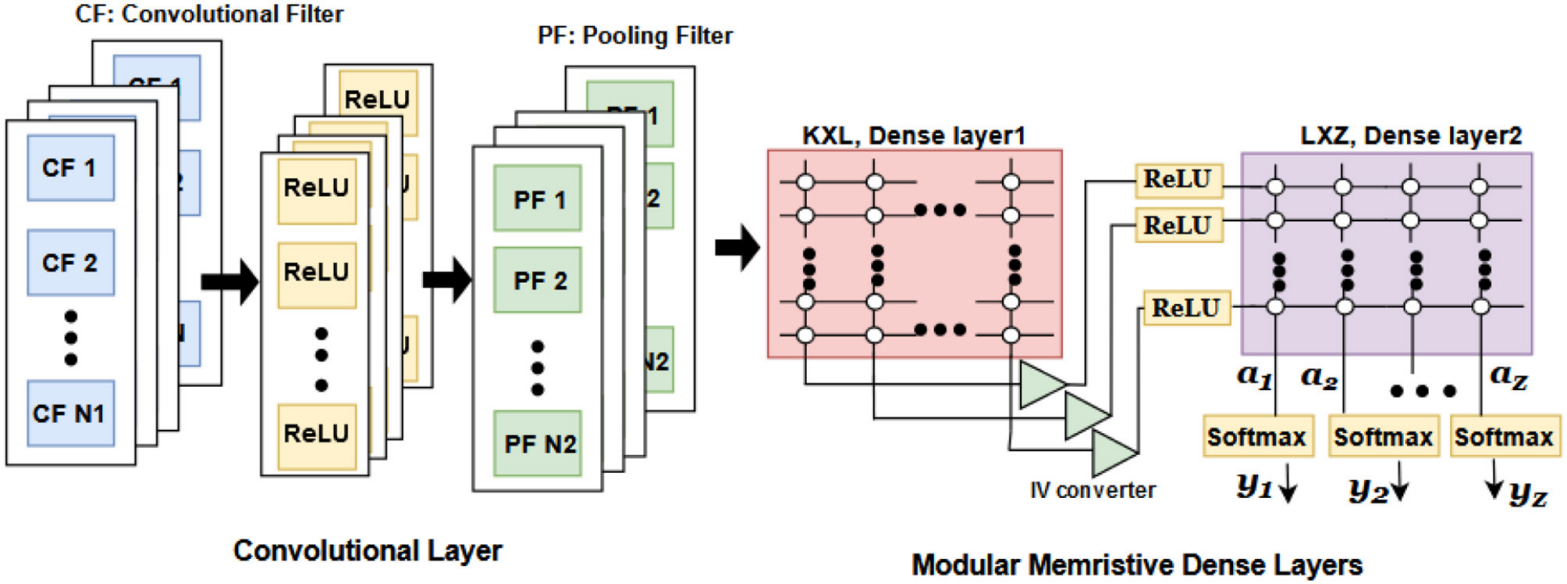
CNN implementation using memristor crossbar arrays (R et al., 2022).

There are several analog memristive crossbar implementations of CNN architecture (R et al., 2022). Figure 5 shows the hardware implementation of CNN consisting of a convolution layer, mean pooling layer, and dense layers. The convolution filters are realized as memristive crossbars. The conductance of memristive devices is the trained weights of the convolutional filter (CF). The number of memristors in each layer is determined by the required feature maps. The features are then fed to the pooling layer circuit. The pooling layer reduces the dimensionality by performing mean-pooling operation (R et al., 2022). The output of the mean-pool operation is flattened and is connected to dense layers for classification. The current-to-voltage (IV) converter block is used to convert currents to corresponding voltages. The activation functions used are ReLU (rectified linear unit) and softmax.

#### 2.3.3. Cellular neural network (CeNN)

**Figure 6:**
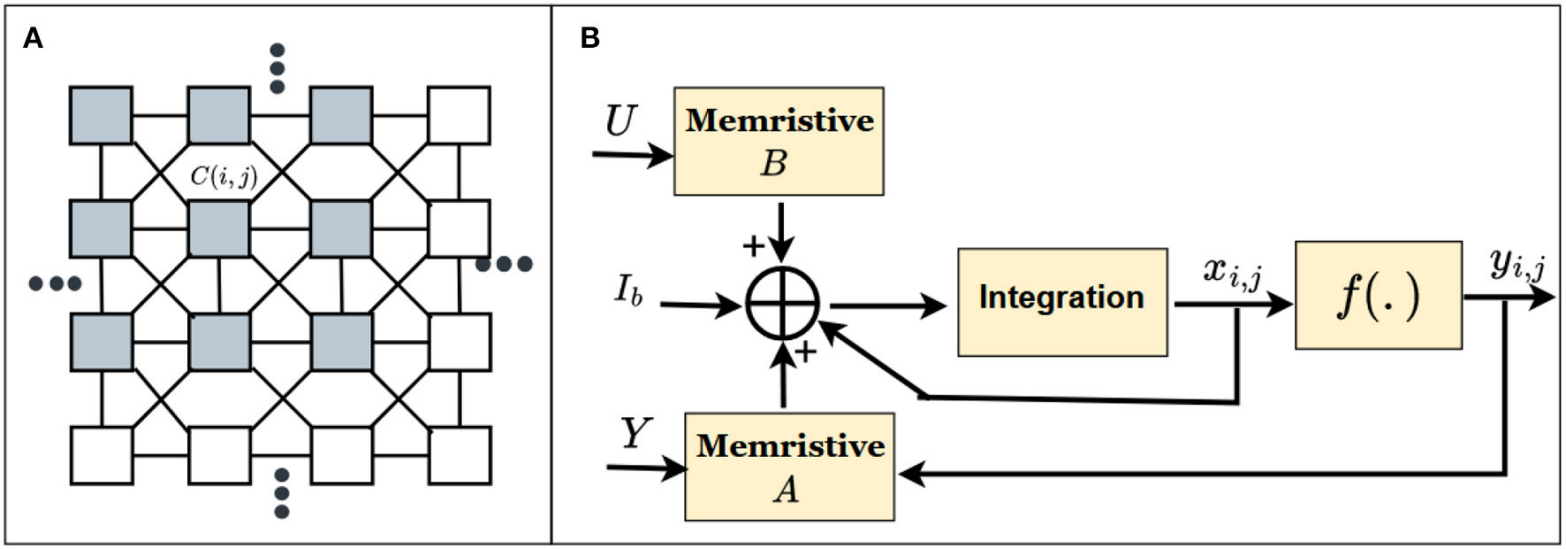
**(A)** Structure of CeNN and **(B)** CeNN implementation using memristor crossbar array (Hu et al., 2016).

The CeNN is developed by Chua and Yang by mimicking the features of neural networks and cellular automata finds applications in the area of image processing (Chua and Yang, 1988a,b). The CeNN network in Figure 6 consists of *I* × *J* cells. Each cell is connected only to its neighboring cells. The connections from each cell *C*(*i, j*) to its neighbors is defined by cloning templates, *A*(*i, j*; *k, l*) and *B*(*i, j*; *k, l*), for feedback and feedforward connections (Chua and Yang, 1988b; Duan et al., 2015). The input signal *U* is connected to *C*(*i, j*) through the feedforward weights *B*(*i, j*; *k, l*). The output of the cell *y*_*k, l*_ is fed to *C*(*i, j*) through the feedback weights *A*(*i, j*; *k, l*). The state equation can be mathematically expressed as Chua and Yang (1988b).


(1) $$\begin{array}{l} \frac{\text{d}{\text{x}}_{\text{i},\text{j}}}{\text{d}t}=-x_{i,j}+\underset{c_{k,l}}{\sum }A\left(i,j;k,l\right)y_{k,l}+\underset{c_{k,l}}{\sum }B\left(i,j;k,l\right)u_{k,l}+I_{b}, \end{array}$$

where *I*_*b*_ is the bias current, *x*_*i, j*_ is the cell state, and *y*_*i, j*_ is the output, respectively. There are various memristive implementations of CeNN in the literature (Duan et al., 2015; Hu et al., 2016). In Figure 6, the feedback and feedforward connections in the CeNN network are implemented using memristor crossbar arrays.

#### 2.3.4. Recurrent neural network (RNN)

**Figure 7:**
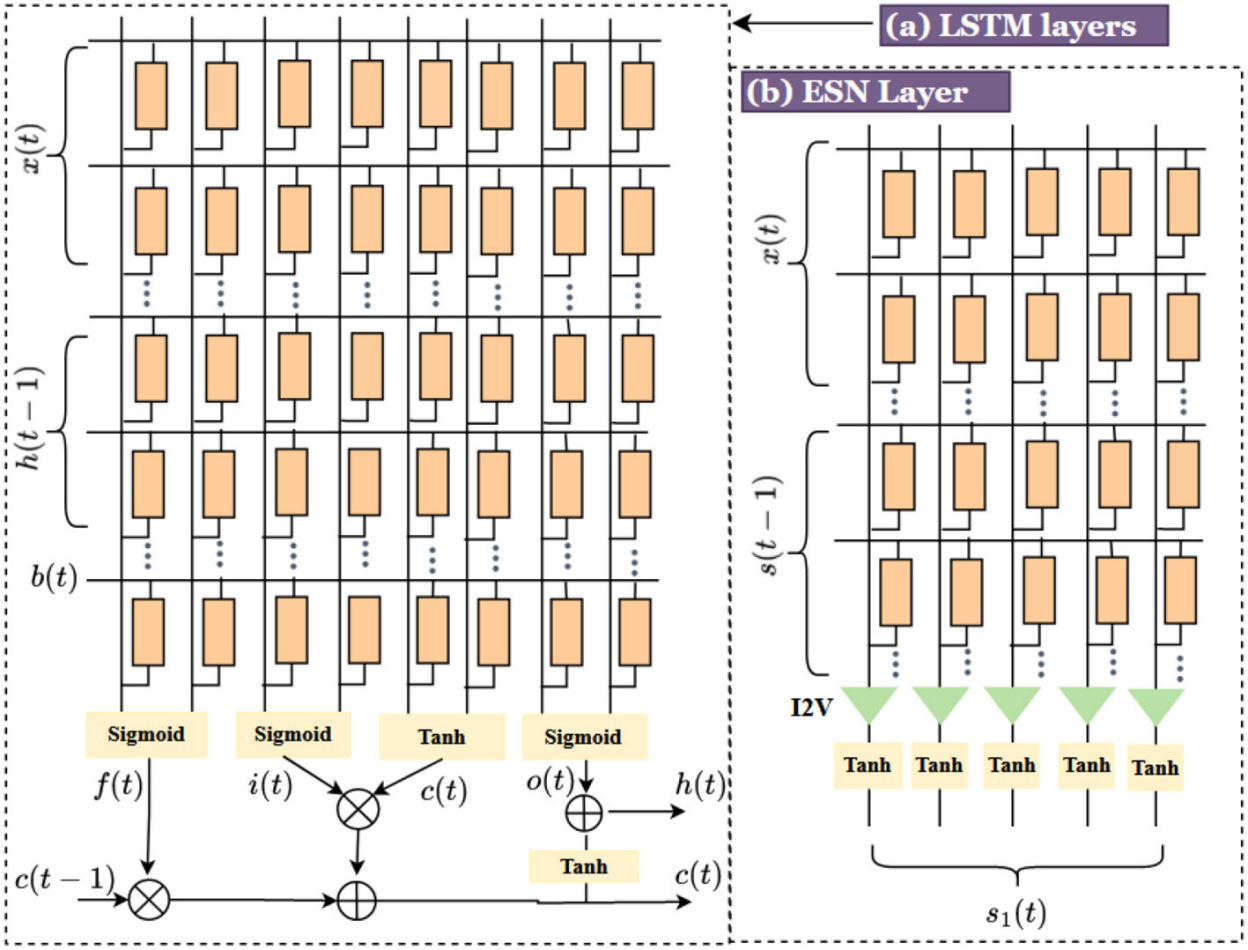
**(a)** Memristive crossbar LSTM architecture (Adam et al., 2018), **(b)** ESN architecture.

The recurrent neural networks-based methods demonstrated outstanding ability in prediction tasks using time-series data by combining large dynamical memory and adaptable computational capabilities. Long short-term memory (LSTM), the special configuration of RNN, is aimed at overcoming the vanishing gradient problems in conventional RNN (Adam et al., 2018). The memristive hardware implementation is presented in Figure 7A (Adam et al., 2018). The input data to the network is the concatenation of input data *x*_*t*_, data from previous cell *h*_*t*−1_ and *b*_*t*_. The input is multiplied by a weight matrix which is the programmed conductance value of the memristor crossbar array. The crossbar outputs are the input to the activation functions (either sigmoid or hyperbolic tangent) to get the gate values. *f*_*t*_ is the output value of forget gate, *i*(*t*) is the output of input/update gate, *o*(*t*) denotes the output from the output gate, and *c*(*t*) denotes the cell state.

The calculation time in LSTM is very heavy and time-consuming. Echo state network (ESN), a reservoir computing architecture, has emerged as an alternative to the gradient descent training method for RNN (Yu et al., 2022). ESN consists of an input layer where the inputs are associated with a weight matrix *w*_*in*_, followed by a recurrent and sparsely connected reservoir using weight matrix *w*_*res*_ and finally, a readout layer associated with a weight matrix *w*_*out*_. The memristive architecture of the ESN reservoir layer is shown in Figure 7B. In ESN, the output readout layer is only trained, and the input and reservoir weight matrices are randomly generated and fixed throughout. The input weights are sampled from a uniform distribution *u*(−*a, a*), using a scaling factor *a* and not trained. The weights of the reservoir are sampled from *u*(−1, 1). Hence, the ESNs are conceptually simple and practically easy to implement.

#### 2.3.5. Spiking neural network (SNN)

**Figure 8:**
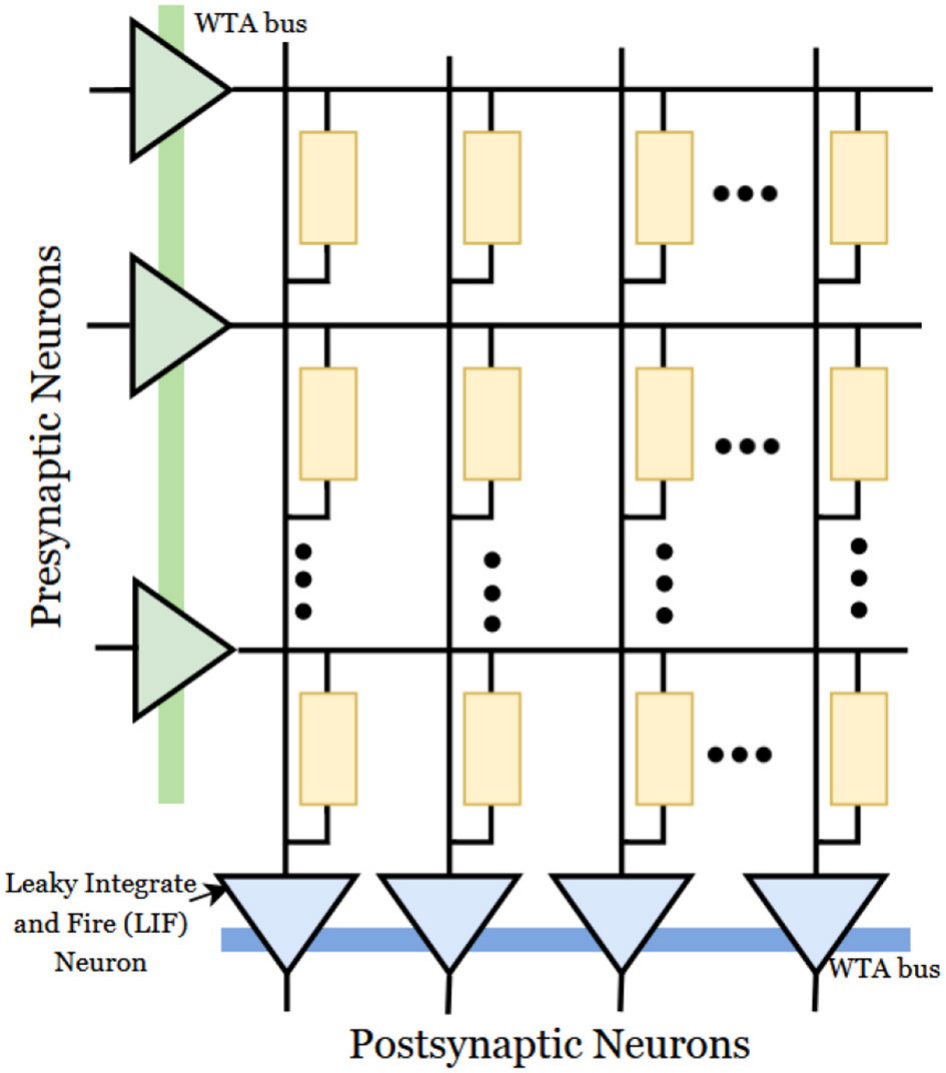
Memristive spiking neural network (Wu et al., 2015b).

The main advantage of SNN hardware implementation is reduced power dissipation in comparison with the pulse-based systems. The data signals are transmitted as spikes in SNN. The SNN is based on the emulation of brain processing using particular spike events represented by spike-timing-dependent plasticity (STDP). STDP is based on presynaptic and postsynaptic impulses. The implementation of SNN architectures with STDP using memristive crossbar arrays is presented in Figure 8. The architecture consists of presynaptic and postsynaptic neurons connected through memristor crossbar arrays. Most cases use a winner-takes-all (WTA) approach for implementation (Wu et al., 2015b). Recent studies introduce stochasticity by adding noise to WTA architecture (Bill and Legenstein, 2014; Krestinskaya et al., 2020). Stochasticity introduces the biological concept of the probabilistic behavior of neurons in the brain.

As discussed in the section, the field of neuromorphic computing using memristor crossbar arrays is advancing and the exploration of novel materials and devices for in-memory computing is required to improve efficiency and scalability. The RRAM devices are promising candidates for synapses and neurons in neuromorphic circuits. The analog tunable capability of RRAM devices enables novel computing functions for the realization of neuromorphic computing. The material class for RRAM devices is from magnetic alloys, metal oxides, 2D materials, and organic materials. Existing studies in the literature report that 2D material-based RRAM devices have better properties compared to conventional electrode materials which enhances the characteristics of RRAM in such a way to improve its application in neural computing. The coming section reviews the mechanism of the working principle of RRAM and the use of 2D materials for enhancing the properties are discussed in detail.

## 3. Graphene and 2D materials based RRAM for neural computing

Graphene and other 2D materials have the potential to revolutionize neural computing due to their unique electrical, mechanical, and optical properties. Graphene is a single layer of carbon atoms arranged in a hexagonal lattice, and it is a highly conductive and transparent material. Other 2D materials, such as transition metal dichalcogenides (TMDs) and hexagonal boron nitride (h-BN), also exhibit interesting properties that make them promising for use in neural computing (Zhang et al., 2022).

TMDs have gained significant attention in recent years due to their unique properties and potential applications in various fields, including neural computing. TMDs are a class of materials composed of transition metals (such as molybdenum or tungsten) and chalcogen elements (such as sulfur or selenium). TMDs can be used to create synaptic devices, which are fundamental building blocks of artificial neural networks (Cao et al., 2021). TMDs exhibit excellent electrochemical properties, allowing them to function as efficient and reliable synapses. By controlling the electrical current through TMD-based synaptic devices, the strength of synaptic connections can be modulated, mimicking the synaptic plasticity observed in biological neural networks (Sung et al., 2022). TMDs can also be utilized in the development of neuromorphic computing systems. These systems offer advantages such as parallel processing, low power consumption, and efficient data processing (Lu et al., 2023). TMD-based devices can be integrated into neuromorphic architectures to perform tasks such as pattern recognition, data analysis, and decision-making (Ko et al., 2020).

Another 2D material suitable for neural computing is h-BN (Xie et al., 2022). h-BN is a two-dimensional material, similar to graphene, but with insulating properties. It can serve as a platform for fabricating electronic components, such as transistors, interconnects, resistive memory, and sensors, with potential applications in neural computing. h-BN has been explored as a material for developing neuromorphic devices that can emulate the behavior of biological neurons. The two-dimensional nature of h-BN allows for the integration of multiple components into compact and efficient architectures.

Graphene-based electrodes have been shown to be biocompatible and capable of recording neural signals with high resolution and sensitivity. Additionally, graphene-based transistors have demonstrated fast switching speeds and low power consumption, making them suitable for use in neural signal processing. Another potential application is in the development of neuromorphic computing, which aims to mimic the structure and function of the human brain (Schranghamer et al., 2020). Graphene and other 2D materials can be used to create artificial synapses, which are the connections between neurons that allow them to communicate with each other. The details of fabrication techniques and applications of 2D materials are shown in Table 1.

**Table 1:** Review on 2D materials for neuromorphic computing applications.

**Sl no**.	**References**	**2D Material**	**Fabrication method**	**Target application**	**switching voltage**
1	Schranghamer et al. (2020)	Graphene	Chemical vapor deposition (CVD)	High precision neuromorphic computing	5.5 V
2	Qian et al. (2016)	h-BN	CVD	Resistive memory	0.72 V
3	Xu et al. (2019a)	MoS_2_	MOCVD	Synapse	0.2 V
4	Kumar et al. (2019)	WS_2_	RF sputtering	Memristors	1.6 V
5	Krishnaprasad et al. (2019)	MoS_2_/ Graphene	CVD	Synapse	1V
6	Liu et al. (2012)	MoS_2_/r-Graphene oxide	Liquid exfoliation	Resistive memory	3.5 V

Overall, graphene and other 2D materials and their combinations hold a great promise for advancing the field of neural computing and could lead to the development of more efficient and powerful neural interfaces and neuromorphic computing systems. Among the 2D materials, the present review focuses mainly on the role of graphene and graphene oxide for RRAM for application in neural computing. There are still many challenges to overcome, such as improving the scalability and reproducibility of these materials and devices, before they can be widely adopted in practical applications (Lin et al., 2016). In this section, the importance and synthesis methods of graphene are discussed in brief and a detailed analysis on the structure and working principles of RRAM is included for a better understanding of the applications of graphene-based RRAM in neural computing.

### 3.1. Properties of graphene and the different methods for its synthesis

**Figure 9:**
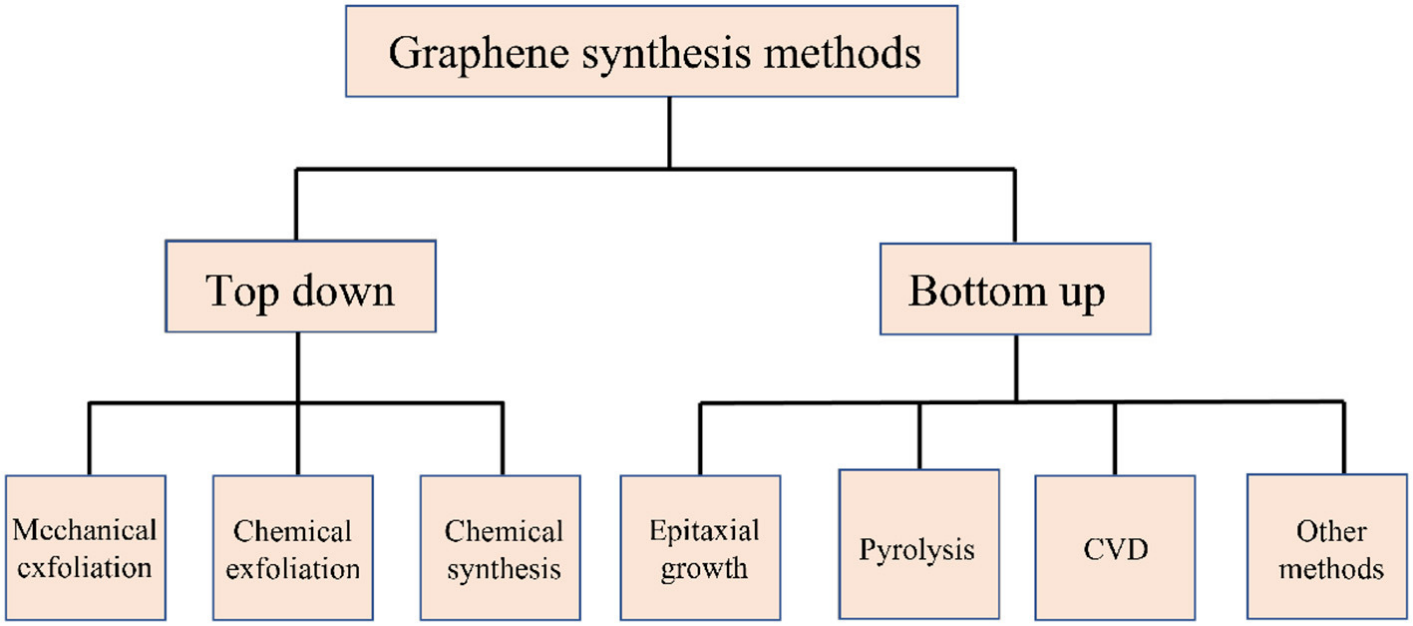
Schematic representation of different methods of graphene synthesis.

Graphene is a 2D material made up of a single layer of sp^2^ hybridized carbon atoms, arranged in a hexagonal lattice. The one atomic layer thickness makes graphene lightweight and flexible. The strong atomic bonding with the nearest carbon atoms provides high mechanical strength to the system, greater than that of steel. Many of these properties vary based on the quality of graphene synthesized. Figure 9 shows the classification of graphene synthesis methods prevalent today. The most popular approaches include those as follows:

1. Chemical vapor deposition (CVD) - The copper or nickel substrate is heated in a reactor chamber while introducing a hydrocarbon gas (such as methane) to the chamber. These hydrocarbons react with the substrate to form graphene.
2. Epitaxial growth - Substrates similar to crystal structure of graphene [e.g., silicon carbide (SiC) or hexagonal boron nitride (h-BN)] can be used to grow graphene for obtaining epitaxial growth via CVD process.
3. Mechanical exfoliation - The bulk crystal graphite consists of multiple layers of graphene. These layers are peeled off using tape or a sharp object.
4. Electrochemical exfoliation - The electrolyte solution is used to exfoliate graphene from graphite.
5. Solvothermal synthesis - The exfoliation of graphene from a bulk crystal of graphite is done in an autoclave having high pressure and temperature.
6. Thermal reduction of graphene oxide - The repeated reduction of graphene oxide by heating in a hydrogen gas environment can result in graphene formation.

The discovery of graphene was through the mechanical exfoliation (Novoselov et al., 2004) of graphite. Different exfoliation techniques such as mechanical exfoliation, liquid-phase exfoliation (Nicolosi et al., 2013; Farajian et al., 2019), and electrochemical exfoliation (Chen et al., 2019; Ejigu et al., 2019) are used for the synthesis of graphene. In the case of mechanical exfoliation of graphene, highly ordered pyrolytic Graphite (HOPG) is used. The simplest method to exfoliate is by using a scotch tape, and the graphene layer is transferred to the required substrate by sticking the tape on it. However, large-scale synthesis of graphene through this approach is time-consuming, expensive, and not practical. In practice, the use of CVD is more commonly used to obtain high-quality graphene films (Fujita et al., 2017). In the CVD process, the gaseous reactants combine to produce the graphene layer on the substrate surface. Depending on the substrate temperature, the formation process of the sample can be controlled. With the CVD process, relatively high-quality graphene can be produced. The modern CVD techniques can be classified into LPCVD (low-pressure CVD) and UHVCVD (ultrahigh vacuum CVD) (replace with PECVD, hot wall, and cold wall) (Mueller et al., 2014; Sharma et al., 2020).

In CVD, the deposition of a monolayer graphene on the surface of a metal substrate is relatively easy and has a large area scalability potential. Several other growth techniques have been reported for graphene synthesis toward RRAM applications including atomic layer deposition (ALD) (Zhang et al., 2014a), solution deposition techniques (Zhong et al., 2015), plasma-assisted techniques, reduction of graphene oxide (Kurian, 2021), arc discharge (Li et al., 2011). Solution coating methods such as spin coating (Long et al., 2019), dip coating (Kim et al., 2019), and drop coating (Puah et al., 2020) offer attractive platforms for obtaining high-quality graphene films due to their low-cost and large area processability. Laser scribing technology can be used to convert GO to rGO using laser, and RRAM realized using laser scribed reduced graphene oxide was reported in Li et al. (2016). CO_2_ laser-induced graphene (LIG) can be used for the fabrication of RRAM, where the graphene is transferred to polydimethylsiloxane (PDMS) from polyimide (PI) (Jung et al., 2021) and SnO_2_ is deposited on it. This will provide a flexible RRAM device. Graphene is the thinnest material discovered to date, and properties such as transparency, and flexibility make this suitable for various electronic device applications.

### 3.2. Features and working mechanisms of RRAM

RRAM is a non-volatile memory that makes use of a material sandwiched between two metal electrodes that have resistive switching properties. The resistance of the RRAM changes depending on the voltage applied across it.

The popular resistive switching material such as titanium dioxide (TiO_2_) resistance can be changed by the application of electrical current to the RRAM. The change in resistance to a high or low resistance is mapped to binary states of “0” and “1”, thereby allowing digital storage. By applying voltage pulses to the RRAM electrode resistance of the TiO_2_ film can be changed. The change in resistance is dependent on the frequency as well as the amplitude of the pulses applied. The RRAM can be read by applying a small voltage pulse and reading the output currents without disturbing the resistance state.

**Figure 10:**
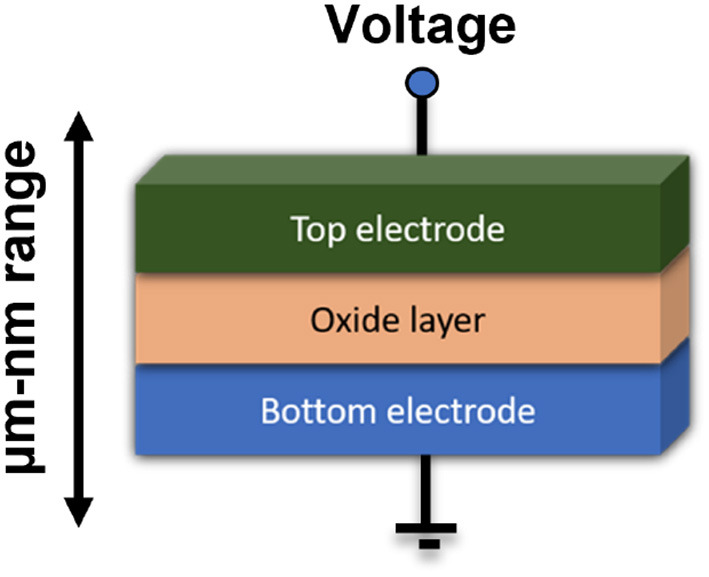
Schematic structure of RRAM with metal-insulator-metal layer structure.

The MIM layer format is used to create the structure of RRAM as shown in Figure 10. The resistive switching mechanism is enabled with applications of voltage across the two terminals of RRAM to define the resistive state. The HRS is considered the OFF state, and the LRS is regarded as the ON state. The switching mechanism from HRS to LRS happens through the application of external voltage. Some of the materials which exhibit this switching include the oxides of hafnium (Long et al., 2013; Zhao et al., 2014b; Feng et al., 2016), titanium (Yang et al., 2009; Bousoulas et al., 2016), tantalum (Chiu et al., 2012; Prakash et al., 2015; Huang et al., 2016), zinc, nickel (Lee et al., 2008b), manganese (Zhang et al., 2009), magnesium (Chiu et al., 2012), aluminum (Wu et al., 2010), and zirconium (Lin et al., 2007; Wang et al., 2009). In RRAM, the choice of electrode material is critical since it affects the switching property of the system. A small read voltage is applied to understand the system's current state (either ON or OFF) without disturbing the system's state. Since RRAM is a non-volatile memory, it will preserve the state even after removing the external voltage.

RRAM can be classified into two types depending on the voltage polarity to unipolar and bipolar resistive switching. The RRAM is unipolar when the used voltage polarity is the same, and it is called bipolar if reverse voltage polarity is used for switching between the different resistance states (LRS and HRS).

**Figure 11:**
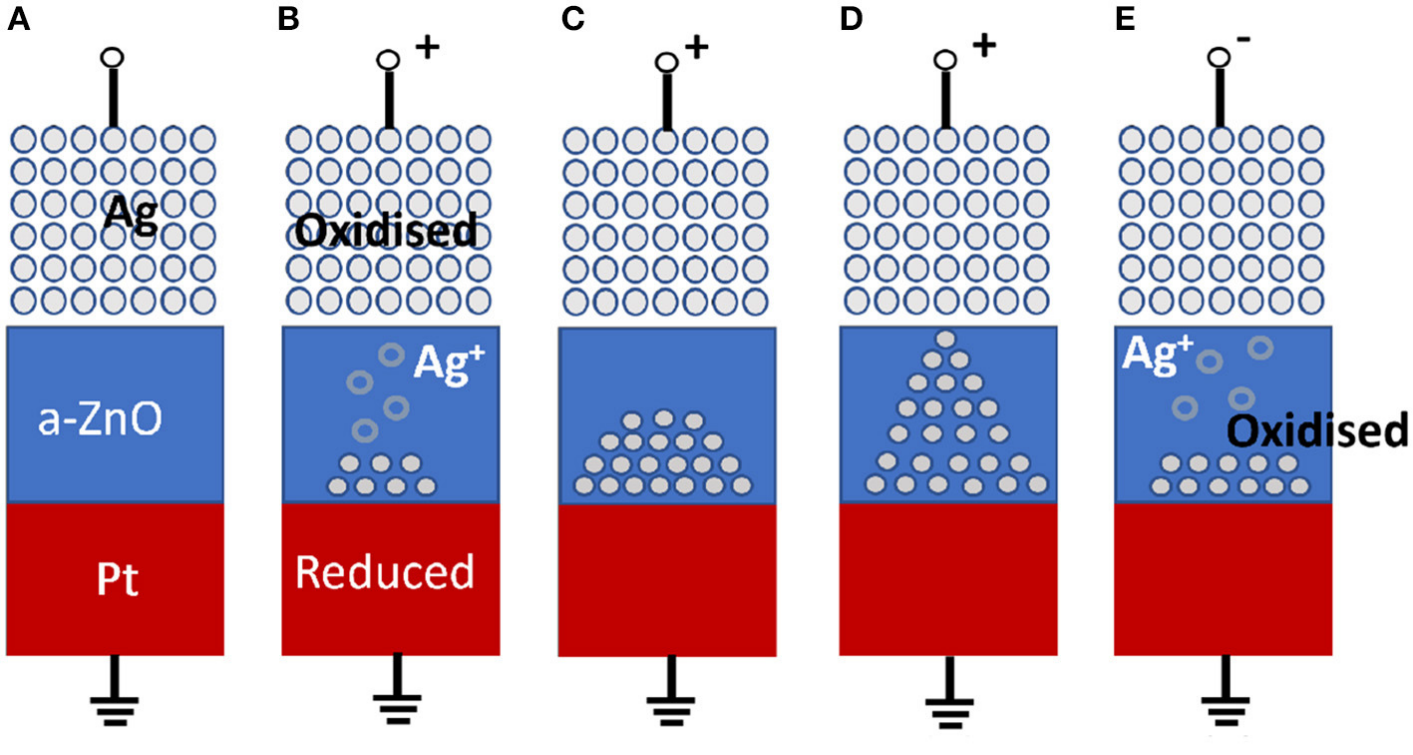
Schematic of the switching mechanism of conductive bridge RRAM. **(A)** The pristine state of the RRAM device. **(B, C)** Oxidation of Ag and migration of Ag+ cations toward the cathode and their reduction. **(D)** Accumulating Ag atoms and Pt electrodes leads to the growth of highly conductive filaments. **(E)** Filament dissolution takes place by applying a voltage of opposite polarity (Zahoor et al., 2020).

The insulating and conducting mechanisms in the RRAM occur from the breakdown and growth of the filament on the application of an external voltage. Depending on the resistive mechanism, RRAM can be classified into (i) metal ion-based RRAM and (ii) oxygen vacancies filament-based RRAM. In metal ion-based RRAM, the switching mechanism happens by the migration of metal ions in the filament and the oxidation and reduction mechanism. The steps followed in the process of transitioning of conducting state to the insulating state are depicted in Figure 11.

This type of mechanism happens in the case of metal electrodes such as Au, Ni, or Cu at the top-level electrode. The migration of metal ions occurs through the dielectric layer, and the subsequent reduction or oxidation happens at the bottom. This will create a metal filament between the two metal electrodes through the dielectric barrier. This metal filament formation possesses the LRS state, and the disappearance of the same enables the HRS state. In Figure 11, the Ag/a-ZnO/Pt RRAM cells demonstrate the resistive switching mechanism. In this case, the Ag electrode is the active element that takes part in the filament formation mechanism, and the Pt electrode is inert. The state of the RRAM devise in the absence of an external electric field is shown in Figure 11A. On applying an external voltage, the oxidation of silver takes place, and it starts to get deposited on the dielectric layer. The bottom electrode, having a negative polarity, will attract these ions, and the ions get deposited on the bottom layer. The formation of metal filament through this process puts the device in the LRS state, as shown in Figures 11B–D. The device can be switched to the HRS state by applying the voltage in the reverse direction, as shown in Figure 11E. We can use graphene as a top/bottom electrode as well as an active insulating layer instead of other materials, as discussed in the following section.

In the case of oxygen vacancies-based RRAM, the resistance-switching mechanism occurs with the creation of oxygen vacancies. The reaction of oxygen ions with the anode material will create the conducting filament. The properties of RRAM will depend on the type of materials present in the top electrode, bottom electrode, and middle layer. Different substitutions of the top and bottom electrodes and middle layers with different materials can enhance the properties of RRAM. The use of 2D materials has shown an enhancement in endurance, switching speed, threshold voltage, retention time, etc. The graphene-based RRAM shows promising results in the modification of RRAM toward better performance and for making the system a multilevel cell storage device for the application of MAC computing.

Different parameters will affect the performance of the RRAM device. This study mainly focuses on the variability-averse multi-level cell storage in the graphene-based RRAM system. The RRAM devices have shown a large variability due to the stochastic nature of the switching process.

## 4. Graphene-based RRAM

Improving the reliability, scalability, and cost-effectiveness of the RRAM device is an essential requirement for practically realizing in-memory and neural computing applications. Graphene-based RRAMs (GRRAM) have different characteristics: low power consumption, higher density, transparency, and homogeneity. GRRAM can be divided into two sub-parts: graphene RRAM and graphene oxide (GO) /reduced graphene oxide (rGO) RRAM. In graphene RRAM, graphene is used as an electrode, whereas in graphene oxide/reduced graphene oxide RRAM, GO or rGO can either be used as a dielectric layer or electrode to enhance the device's performance.

### 4.1. Graphene as the electrode in RRAM

The main property of RRAM is the resistive switching mechanism which has various difficulties related to the selection of electrodes and the dielectric layer. The high conductivity and high surface area-to-volume ratio of graphene makes it suitable for electrodes. The power consumption is significantly less in graphene-based electrodes in RRAM compared to conventional metal electrodes in RRAM memory devices. Graphene as an electrode offers various advantages over traditional metal electrodes. The greater mechanical scalability, higher conductivity, and ultrathin nature of graphene help to design non-volatile RRAM memory devices. The mechanical properties of graphene, including exceptional strength, flexibility, and elasticity, make it an ideal candidate for use in RRAM devices. These properties enable the fabrication of ultrathin memory cells and provide the potential for integrating RRAM into complex, multi-layered device architectures (Novoselov et al., 2005; Zhang, 2015). The mechanical scalability of graphene allows for the creation of densely packed memory arrays, contributing to higher storage capacities and improved device performance (Papageorgiou et al., 2017). Furthermore, graphene exhibits exceptional electrical conductivity due to its unique electronic band structure (Yung et al., 2013). The switching mechanism in RRAM involves the controlled migration of ions within the memory cell, leading to changes in resistive states. Graphene's high conductivity facilitates efficient charge transport during these switching processes, resulting in fast and reliable switching. The high conductivity of graphene also helps reduce power consumption and enables high-speed read and write operations in RRAM devices.

Lee et al. (2010) report a detailed study on resistive switching characteristics of non-volatile memory devices with nano-materials. 2D material and nanomaterial are the extreme candidates in the nano industry where organic channels and metal electrodes decrease the transmittance value (transmittance decrease of 25%) of the memory devices (Lee et al., 2010). Graphene is used as electrodes, and single-wall carbon nano-tube (SW CNT) is assumed as active layers between metals in non-volatile memory devices. They implemented this memory device with ozone treatment as graphene and oxygen atoms are bonded together. The fabricated memory device revealed that it provides an acceptable transmittance value. Graphene as an electrode provides a minimum decrease of transmittance of 3.6 %, which is 11.4 % and 25 % in Au and Al. They discovered that the non-volatile memory device with graphene electrodes exhibits better conduction with high mobility of 44cm^2^*V*^−1^*s*^−1^ and a switching speed of 100 ns. The graphene-based memory device performs better than metallic electrodes such as Au, Al, and Ag. The graphene SWCNT memory device improves switching characteristics enhanced by 2 × 10^2^ (Yu et al., 2011b).

Ji et al. (2011) approached a design to integrate an 8 × 8 crossbar array of organic memory devices with graphene. This multi-layer graphene is an intermediate layer between insulating polyamide (IP) layers. A fabrication process integrates this device with the help of PET (polyethylene terephthalate) substrate. This device offers a high switching ratio current of 10^6^ with write-once-read-many (WORM) characteristics. The bending cycle is 10 orders larger (Lee et al., 2010) and exhibits excellent cell-to-cell uniformity. The retention time of the memory device has been controlled in the order of 10^4^. Their approach has maintained stable and reliable device characteristics without degrading the current performance. The WORM-type devices store the data permanently without losing any unintended data.

**Figure 12:**
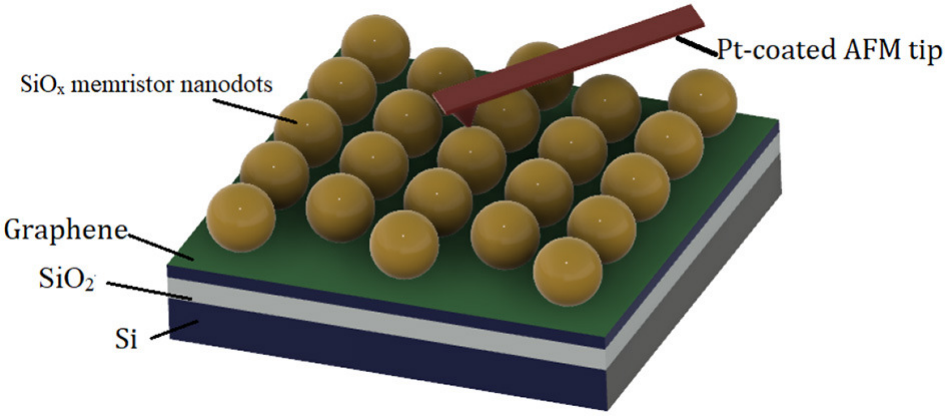
Schematic structure of memristor nanostructures on metal and graphene electrodes by a block copolymer self-assembly process.

Park et al. (2012) demonstrated a detailed fabrication and characterization of high-density memristor nanodots with platinum and graphene electrodes by a block copolymer self-assembly process. Graphene is used as the bottom electrode, and Pt is a top electrode, where silicon dioxide (SiO_2_) is considered an active layer for resistive switches where the memory device has been fabricated with a minimum process cost and less complexity. The fabricated device exhibits a switching ratio of 10^2^, an endurance of 80 voltage sweeps, and a unipolar switching mechanism independent of the supply voltage. The formation of a memristor on a graphene electrode is shown in Figure 12.

**Figure 13:**
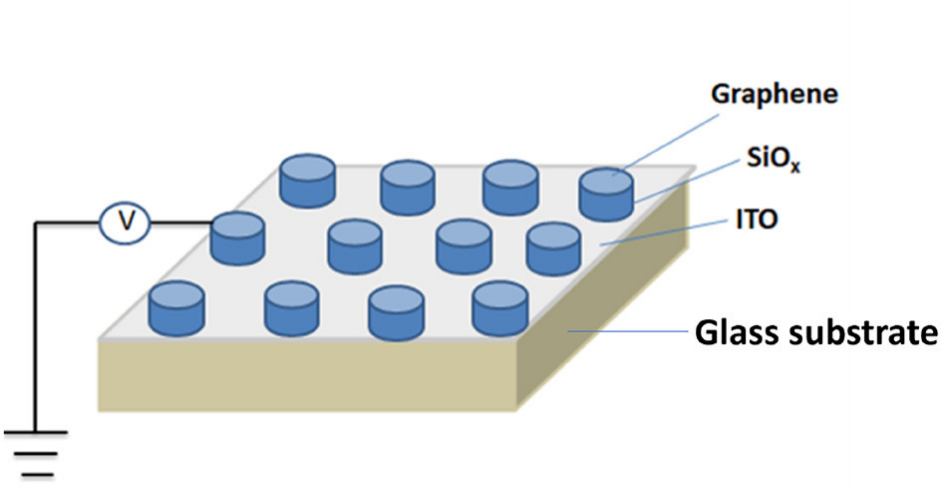
Schematic diagram of graphene-SiO_*x*_ -indium tin oxide (ITO) device.

As transparent electronics devices are in high demand for the electronics industry. Yao et al. (2012) have configured a transparent non-volatile memory device based on SiO_*x*_ active layer, indium tin oxide, and graphene as bottom and top electrodes with the glass substrate. Studies on the various device sizes are pursued to enhance the reliability of non-volatile memory. Their study revealed that the conduction filament generated in SiO_2_ active layer maintains the constant current as the device size increases or decreases. The switching ratio (10^5^) and electrical endurance (300 voltage sweeps) have improved compared to Park et al. (2012). They have also explored how the proposed device with graphene electrode offers better transparency characteristics and low retention time would be beneficial for device application. Figure 13 shows the graphene-SiO_*x*_-indium tin oxide (ITO) device.

**Figure 14:**
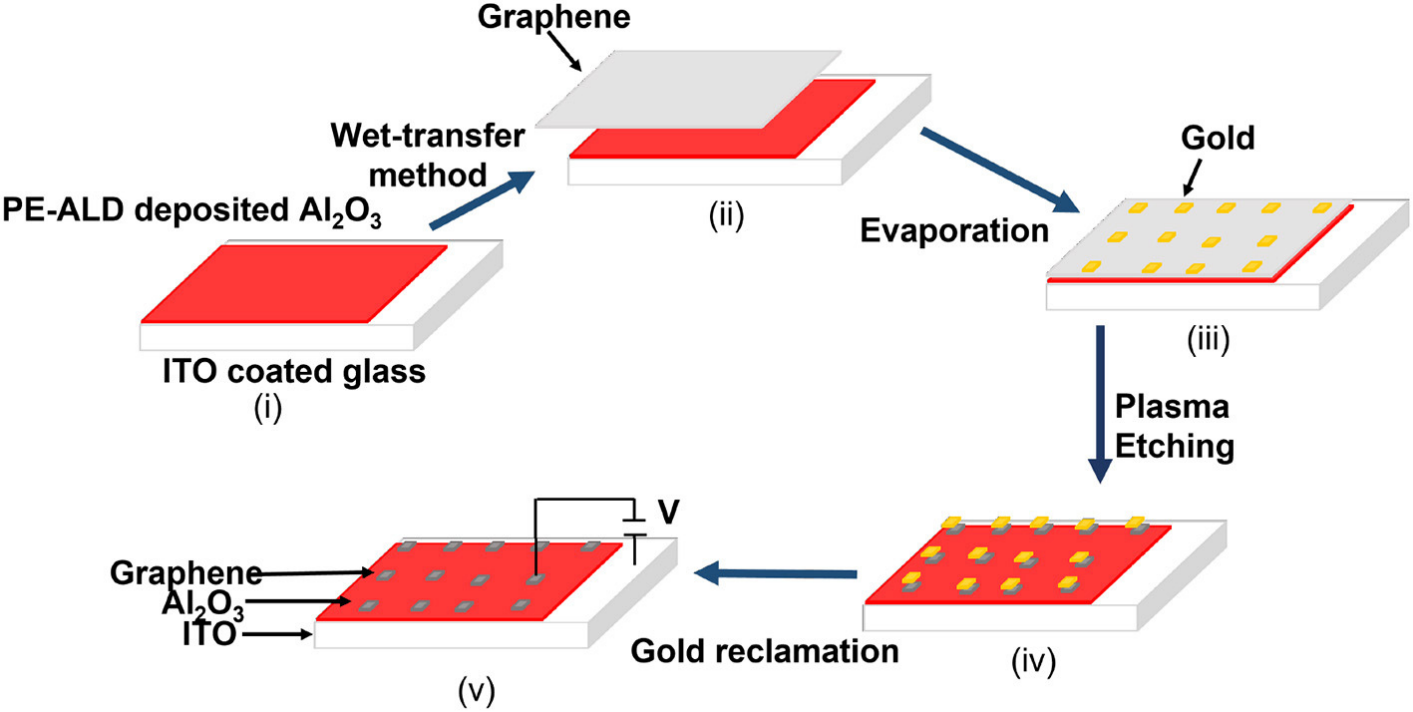
Schematic structure of the Ti/ZrO_2_/Pt RRAM device (Dugu et al., 2018).

A glass platform is a suitable choice for constructing transparent memory devices. The RRAM is constructed with indium tin oxide as the top electrode, alumina as the functional oxide layer, and graphene as the bottom electrode. The non-volatile memory device of this composition has a high transmittance of 82% in the visible region. It is stable and has non-symmetrical bipolar switching properties with low set and reset voltages (less than 1 volt). With its vertical two-terminal configuration, the device has good resistive switching performance and a high on-off ratio (switching ratio) (5 × 10^3^) (Dugu et al., 2018). The figure representing the device structure is shown in Figure 14. Furthermore, transparent materials can be integrated with other optical components to manipulate and direct light within the sensing system. This integration enhances the functionality and performance of optical sensors. Transparent RRAM devices could be integrated with optical sensors, enabling direct interaction between optical input data and neural network processing. This could find applications in fields such as image recognition or computer vision (Zhou et al., 2019; Kalaga et al., 2020).

**Figure 15:**
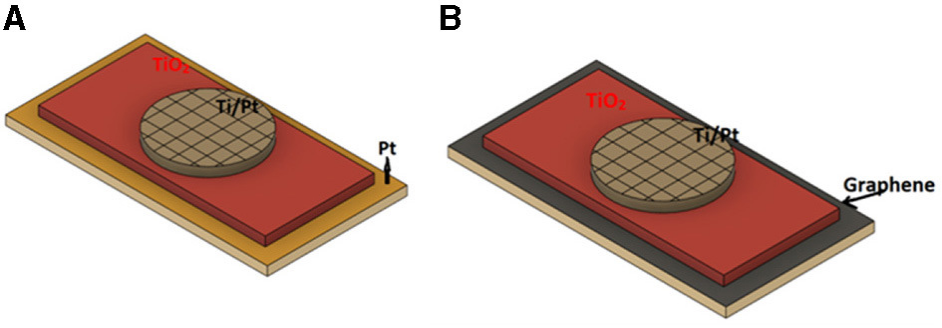
Schematic structure for **(A)** platinum-based memristors devices (PtMDs) and **(B)** graphene-based memristors devices (GMDs).

A graphene-based memristive device (GMD) has been proposed by Qian et al. (2014) and presented a comparative analysis of output performance with a Pt-based memristive device (PtmD). The schematic structure for PtMD and GMDs is shown in Figure 15. The graphene electrode is integrated into TiO_*x*_ by the CVD fabrication method to obtain ultra-low switching power and non-linearity. Unlike Yao et al. (2012), they have used graphene as the bottom electrode, whereas Ti/Pt is used as the top electrode. The GMD is fabricated on polyethylene naphthalate (PEN) and offers excellent retention against mechanical bending. They discovered that GMDs have less switching power compared to PtMDs, which helps to protect the device from any thermal damage. Tunable, ultralow-power switching in memristive devices are enabled by a heterogeneous graphene oxide interface. The summary of RRAM devices graphene as top and bottom electrode along with typical characteristics are listed in Table 2.

**Table 2:** Graphene as the top and bottom electrode.

**References**	**Bottom electrode**	**Top electrode**	**Active layer**	**Substrate**	**Switching ratio**	**Endurance**	**Retention ratio**
Yu et al. (2011b)	Graphene	Graphene	SWCNT	PET	10^3^	-	10^2^
Ji et al. (2011)	Al	Graphene	polyimide and 6-phenyl-C61 butyric acid methyl ester (PI:PCBM)	PET	10^6^	-	10^4^
Park et al. (2012)	Graphene	Pt	SiO_2_	Si	10^2^	80	-
Yao et al. (2012)	ITO	Graphene	SiO_*x*_	Glass	10^5^	300	-
Dugu et al. (2018)	ITO	Graphene	Alumina	Glass	>10^3^	-	-
Chakrabarti et al. (2014)	Graphene	Graphene	TiO-x/Al_2_*O*_3_/*TiO*_2_	-	10^4^	>200	10^4^
Ji et al. (2011)	Graphene	Graphene	SWCNT	PET	10^3^	10^2^	10^3^
Ji et al. (2011)	Graphene	Graphene	ZnO	Si	10^3^	50	-
Chakrabarti et al. (2014)	Graphene	Graphene	TiO-x/Al_2_*O*_3_/*TiO*_2_	-	10^4^	10^2^	10^4^
Yao et al. (2012)	Graphene	Graphene	SiO_*x*_	Plastic	10^6^	10^2^	
Park et al. (2012)	Graphene	SiO_*x*_	Pt	Si	10^2^	80	10^4^
Ying-Chih Lai et al. (2013)	Graphene	Al	PMMA:P3BT	PET	10^5^	10^7^	10^4^

Similar to Qian et al. (2014), Lee et al. (2015) fabricated a graphene SET electrode-RRAM (GS-RRAM ) memory device and compared it with a Pt-RRAM memory device. In this study, a thin monolayer graphene that serves as a SET electrode is considered to make a thin memory cell structure. The graphene SET electrode helps to store (SET) and restore (RESET) oxygen ions during the programming process. They revealed that the proposed model with a graphene edge electrode has a lower SET compliance current, low RESET current, and low programming voltages, where the Pt-RRAM device cannot deal with low programming voltage or current due to degradation issues of the memory window. The efficient ion-storing capability of graphene helps reduce the power consumption 300 times more in Pt-RRAM. Metal oxide-resistive memory using graphene-edge electrodes (Chakrabarti et al., 2014) explored the performance of RRAM, where graphene is used as top and bottom electrodes. The TiO_*x*_/Al_2_O_3_/TiO_2_ dielectric layer is sandwiched between the top and bottom electrodes. The device exhibits forming-free switching characteristics where the device transitions between different resistance states (HRS/LRS) without requiring a separate “forming” process. The forming-free behavior reduces the device complexity and faster the switching process. The proposed device has increases the non-linearity of the current-voltage characteristic with a reduced value of current compliance. When the device exhibits increased non-linearity, the relationship between voltage and current is not linear and more complex and may involve various mechanisms, such as threshold effects, hysteresis, or other non-linear behaviors. This non-linearity can be influenced by factors such as the material properties of the dielectric layer and the electrodes as well as the specific design and operating conditions of the device. A stable retention time of 10^4^s, a switching ratio of 10^4^, and a greater endurance value (>200 cycles) have been obtained for the graphene-insulator-graphene (G-I-G) based RRAM configuration. Sohn *et al*. reported a graphene-based 3D RRAM structure where the oxygen ions originating from HfO_*x*_ migrate toward the graphene layer, where they aggregate to create a conductive filament (Sohn et al., 2015). This filamentary layer exhibits exceptional thinness, primarily attributed to the atomic-thick nature of graphene. This aligns with the switching mechanism observed in HfO_2_ RRAM devices utilizing a top electrode composed of TiN in conjunction with a passive bottom electrode (Yu et al., 2012).

### 4.2. Graphene as the middle layer in RRAM

Other than electrodes, graphene can also be used as a middle layer in GRRAM for optimizing the switching properties. The incorporation of graphene in the middle layer helps the filament growth by generating a local internal field and acts as a trapping site in the RRAM. The graphene middle layer is usually used for multilevel switching. It is reported that graphene flakes when used as a middle layer help trap charge and act as a storage medium.

**Figure 16:**
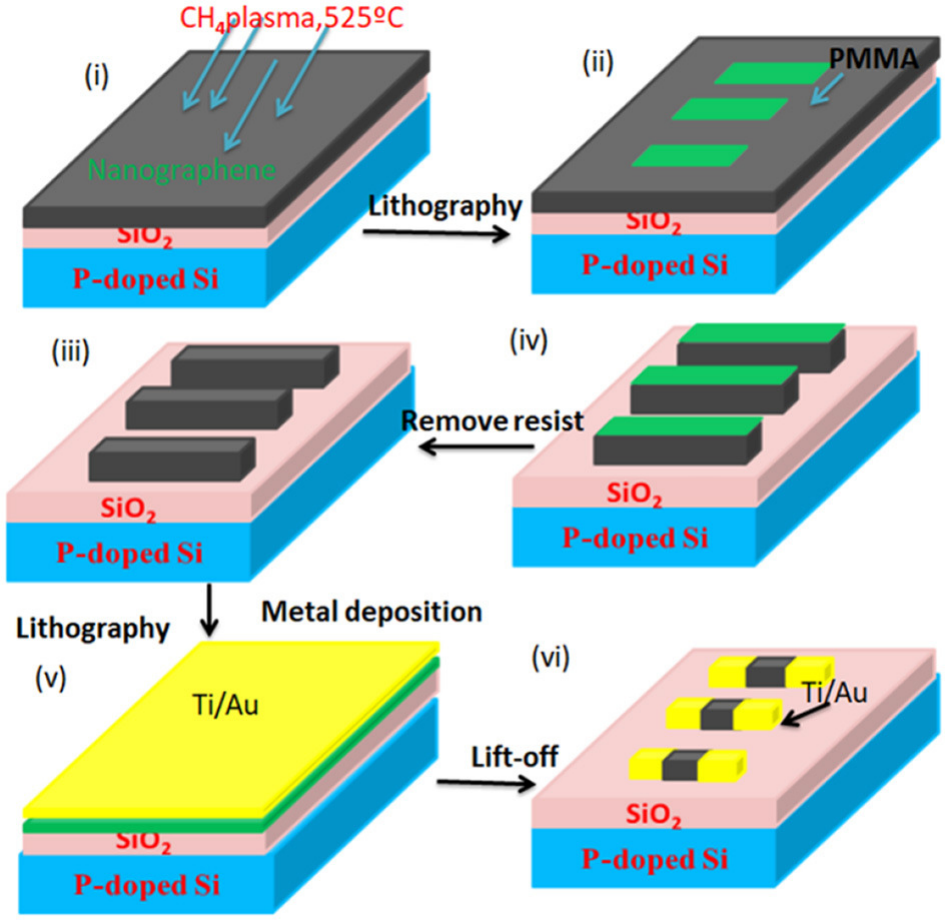
Fabrication flowchart for nanographene (NG).

Doh and Yi (2010) proposed few-layer graphene (FLG) as an active layer in field-effect devices/ferroelectric devices. They studied the effect of the graphene thickness variation to observe the electrical performance. They discovered that the device has bistable resistance characteristics with long retention time. The resistance difference ratio has decreased with the increased value of graphene film thickness. They also demonstrated that power consumption is high due to the high value of operational voltage (V_*G*_ > 30*V*). He et al. (2012) proposed nanographene (NG) which acted as an active layer fabricated on a SiO_2_ substrate. Various multi-level switching mechanisms have been observed, such as unipolar, bipolar, and non-polar characteristics. Nanographene as an active layer in RRAM has several advantages, such as tunable conductivity and an easy fabrication process, unlike other materials. This research has shown a better endurance value of 10^4^ cycles, a faster-switching speed of 500 ns, and a longer retention time of 10^5^ cycles. The fabrication flow chart is shown in Figure 16.

Shindome et al. (2013) experimented with single and multi-layer graphene nanoribbon RRAM device characteristics. The drain current performance has been obtained for changing metal electrodes. They revealed that drain current is more for multi-layer graphene RRAM devices than single-layer graphene RRAM. The research also exhibits lower switching energy with a decreased value of channel width, which increases the packing density of the device. Graphene nanoribbon RRAM can possibly scale down to 30nm. Shin et al. (2010) proposed the charging and discharging effect (CDE) to study the bistable switching effects in graphene devices. They also demonstrate bandgap engineering to improve the switching ratio of the device. Two different charge carriers, p-type and n-type, have been considered for this study. The proposed study revealed that the current hysteresis of p-type graphene is inverted into n-type graphene, which increases the stability of the device. The summary of RRAM devices with graphene as an active layer along with typical characteristics are listed in Table 3.

**Table 3:** Graphene as an active layer.

**Reference**	**Bottom electrode**	**Top electrode**	**Active layer**	**Substrate**	**Switch ing ratio**	**Endurance**	**Retention time**
He et al. (2012)	Ti/Tu	SiO_2_	NanoGraphene	p-doped Si	-	10^4^	10^5^
Shin et al. (2010)	Cr/Au	Al	Graphene	SiO_2_	-	10^2^	-
Shindome et al. (2013)	Ti/Au	Ti/Au	Graphene	SiO_2_	10^3^	10^4^	10^5^
Wu et al. (2012)	ITO	ITO	Graphene	Glass	10^6^	-	10^4^
He et al. (2013)	Ti/Au	Ti/Au	Graphene	Si/ SiO_2_	10^5^	-	-
Shindome et al. (2013)	Ti/Cr/Au	Ti/Cr/Au	Graphene nanoribbon	Si/ SiO_2_	10^6^	10^2^	10^3^

## 5. Graphene oxide (GO)/reduced graphene oxide (rGO) RRAM

Graphene as a two-dimensional crystal has received more attention from researchers in the semiconductor industry due to its ultrahigh mobility, high thermal conductivity, and transparency characteristics. Graphene oxide is a layered structure consisting of a monolayer of graphene bound to oxygen in carboxyl, hydroxyl, or epoxy groups. Having a high energy band-gap of graphene oxide is possible to reduce the energy band-gap by removing the C-O bonds and offers high solubility. Graphene oxide and reduced graphene oxide are the two important carbon materials mainly used in bioelectrochemical systems (BESs). Graphene oxide offers a large hydrophilic surface area with oxygen-containing functional groups, facilitating microbial attachment and tailored electrochemical reactions on its electrode surface (Singh et al., 2018). On the other hand, rGO, obtained from GO through reduction processes, provides enhanced electrical conductivity, improved biocompatibility, and potential catalytic activity, making it an ideal candidate for efficient electron transfer and biofilm formation in BES systems (Wu et al., 2019). Graphene oxide can be deposited on any substrate due to its flexible nature. Nowadays, GO is a good insulating and semiconductor material compared to other materials and is highly used for RRAM devices. Graphene oxide-based RRAM devices have various pros compared to other materials. The RRAM device with GO can be scaled down in nano-regime and increases the packing density due to the easy fabrication process. Hu *et al*. studied graphene oxide (GO) based RRAM device flexible non-volatile memory. For the purpose of this study, aluminum (Al) has been chosen as the top and bottom electrodes, while GO functions as the active layer. When a negative voltage is applied, it induces an electric field that prompts the migration of oxygen ions within the GO layer. This migration leads to the formation of localized conductive filaments (CFs), consequently causing the device to switch to a LRS. Notably, at the LRS, ohmic conduction is not observed due to the transformation of the GO film into a sp3-bonded state in the absence of CFs (Jeong et al., 2010). During the forming process, a positive voltage bias applied to the Al layer initiates the creation of a highly resistive region in proximity to the tunneling electrode (TE). In the presence of an external electric field, oxygen ions present in the dielectric layer migrate toward the electrode. This migration fosters the continuous development of an sp^3^ hybridization layer between the Al electrode and the GO layers that have undergone structural modifications, leading to the high-resistance state (HRS). Subsequently, when a negative voltage bias is applied to the TE Al layer, the reverse diffusion of oxygen ions occurs, resulting in the formation of CFs that lead to the low-resistance state (LRS) near the contact interface, driven by the influence of a negative electric field (Panin et al., 2011). In 2009, He et al. (2009) first explored the RRAM device with graphene oxide (GO) thin films, which are processed by the vacuum filtration method. They found that the device has a low switching voltage and offers a low switching ratio, which is improved later by many researchers (Kim et al., 2011; Yi et al., 2014). Jeong et al. (2010) fabricated a GO-based RRAM device prepared by the spin casting method at room temperature and found to be more reliable and flexible. This study has increased the retention and endurance of the device, which would be helpful for memory applications.

Graphene oxide (GO) can be used for non-volatile and bistable memory devices for its high optical transparency and flexibility. Vasu et al. (2011) studied the unipolar switching effect on reduced graphene oxide (rGO) with the glass substrate to obtain a high switching ratio and switching speed. The obtained results exhibit a switching ratio of 10^5^ and switching speed of 10 *μs*.

Rani et al. (2012) implemented a cost-effective non-volatile memory behavior in rGO memory devices for extracting better endurance and retention time. It is found that the rGO memory device exhibited an endurance value of 10^2^ and a retention time of 10^5^. Ho et al. (2014) demonstrated a comparative analysis between rGO and GO RRAM devices for impedance spectroscopy and current-voltage analysis. The impedance spectroscopy and current-voltage analysis have been studied to determine the possible physical mechanism for resistive switching behavior. It is observed that switching behavior can be noticed in rGO-based RRAM devices due to its oxidation and reduction at the top electrode. The obtained results for rGO were better with the retention time of 10^6^s. However, the rGO memory device provides a large value of the operating voltage of 4V, which increases the power consumption.

Pradhan et al. (2016) proposed a non-volatile rGO-based RRAM memory device to reduce the threshold voltage, which solves the power losses problem of the device more than Ho et al. (2014). Pradhan et al. (2016) proposed an rGO RRAM device which exhibits a threshold value of less than 1V where 4V was achieved by Ho et al. (2014). They also checked the variability of device size, film thickness, and scan votlage.

Kim et al. (2014) demonstrated a transparent memory cell, where reduced graphene is placed between two ITO electrodes to observe the multi-level resistive switching purpose. This memory device offers 80% optical transmittance where the amplitude of applied pulse voltage was varied from 2 to 7V.

Lin et al. (2015) developed a ZnO RRAM device with a capping rGO layer to study the resistive switching behavior. They concluded that introducing the rGO layer increases the stability of the ZnO memory device with a switching ratio of 10^5^. The rGO layer acts as an oxygen reservoir in the ZnO memory device where ions are transit easily. On the other hand, oxygen vacancies of the rGO layer oppose reacting with Al electrodes. They also mentioned that ZnO RRAM device offers a great value of endurance of 10^8^. The summary of RRAM devices with graphene oxide and reduced graphene oxide as an active layer is listed in Table 4.

**Table 4:** Graphene oxide and reduced graphene oxide as an active layer.

**Reference**	**Bottom electrode**	**Top electrode**	**Active layer**	**Substrate**	**Switching ratio**	**Endurance**	**Retention time**
Wu et al. (2014)	Pt	Cu	GO	Ti/SiO_2_/Si	20	10^2^	10^4^
Hong et al. (2010)	ITO	Al	GO	Glass	10^3^	10^2^	10^9^
Jeong et al. (2010)	Al	Al	GO	PET	10^2^	10^2^	10^5^
Wang et al. (2012a)	ITO	Al	GO	Glass		10^3^	10^2^
Hu et al. (2012a)	Pt	Pt	GO	SiO_2_ /Si	10^4^	10^2^	10^5^
Liu et al. (2013)	GO	GO	GO	PET	10^2^	10^3^	10^3^
Wang et al. (2012b)	Pt	Al	GO	Si	10^4^	10^2^	10^3^
Venugopal and Kim (2012)	Ag	Ag	GO	SiO_2_	10	-	10^3^
Wang et al. (2012a)	ITO	Al	GO	PET	10^2^	10^2^	10^4^
Pradhan et al. (2016)	Al	Al	GO	Glass	10^2^	10^2^	10^4^
Banerjee et al. (2015)	ITO	Au	GO	Glass	10	10^2^	-
Wu et al. (2015a)	ITO	ITO	GO	PES	10	-	10^5^
Nagareddy et al. (2017)	Ti/Pt	Ti/Pt	GO	Si/ Si*O*_2_	10^3^	10^4^	10^5^
Kim et al. (2018)	Pt	Pt	rGO	Si/SiO_2_	10^5^	-	-
Saini et al. (2018)	ITO	Al/Au	GO	Glass	10^5^	-	–
Han et al. (2014)	Ag	Au	rGO	PET	10^4^	10^2^	10^5^
Kim et al. (2014)	ITO	ITO	rGO	Glass	10^3^	10^5^	10^7^

## 6. Comparison of the properties of graphene-based materials with other 2D materials

In sections 4 and 5, the details of graphene, graphene oxide, and reduced graphene oxide base RRAM and its characteristics are discussed. There are other 2D materials such as transition metal dichalcogenides (TMDs) (molybdenum disulfide (MoS_2_) and tungsten diselenide (WSe_2_) etc.), which offer a diverse range of electronic properties as discussed in section 3. TMDS based RRAM is an emerging technology in the field of non-volatile memory and nanoelectronics (Zhu et al., 2019). In TMD-based RRAM, a thin layer of TMD material is used as the switching medium between two electrodes. The resistance of this TMD layer can be altered by applying an electric field, which changes the oxidation state or defects in the TMD material (Zhang et al., 2018; Jian et al., 2022). However, there are also challenges to overcome, such as ensuring stable and reliable switching behavior, understanding the underlying mechanisms that control resistance switching, and developing scalable manufacturing processes (Zhang et al., 2018). Table 5 presents the comparative study of different properties of graphene and TMDs based RRAM. TMDs can form stable heterostructures with graphene, combining the strengths of both materials for various functionalities.

**Table 5:** Comparative analysis of graphene-based RRAM and 2D TMDC materials-based RRAM devices.

**Device name**	**Reference**	**Bottom electrode**	**Top electrode**	**Active layer material**	**Substrate**	**Switching speed**	**Endurance**	**Retention time**
Graphene oxide based RRAM	Wu et al. (2014)	Pt	Cu	GO	Ti/SiO_2_ /Si	20	10^2^	10^4^
	Liu et al. (2013)	ITO	GO	GO	PET	10^2^	10^3^	10^3^
	Nagareddy et al. (2017)	Ti/Pt	Ti/Pt	GO	Si/SiO_2_	10^3^	10^4^	10^5^
	Wang et al. (2012b)	Pt	Al	GO	Si	10^2^	10^2^	10^3^
	Sun et al. (2015)	FTO	Ag	MoS_2_	Glass	10^3^	-	10^2^
2D TMDs based RRAM (MoS_2_, WS_2_, MoSe_2_ based)	Zhou et al. (2017)	ITO	Ag	MoS_2_	Glass	10^4^	10^2^	10^3^
	Das et al. (2019)	ITO	Al	MoS_2_	Glass	10^2^	10^4^	10^7^
	Kumar et al. (2018)	Ni-Mn-In	Cu	MoS_2_	Si	10^2^	10^2^	10^3^
	Rehman et al. (2017)	Ag	Ag	Ws_2_	PET	10^3^	10^2^	10^5^
	Zhou et al. (2016)	Ag	Ag	MoS_2_	SiO_2_	10^2^	10^2^	10^3^

Metal oxides such as hafnium oxide (HfO_2_) and titanium dioxide (TiO_2_) provide unique electronic properties suitable for different device applications (Meyer et al., 2012). Along with memory, they are used in optoelectronic devices, catalysis, and sensing applications. Metal oxides exhibit resistive switching behavior, which makes them suitable for RRAM applications, where the metal oxide layer acts as the switching medium (Sawa, 2008). When a voltage is applied across the electrodes, localized changes in the metal oxide's resistance state occur due to various mechanisms, such as the formation and dissolution of conductive filaments or changes in oxygen vacancy concentration (Kumar et al., 2017). One advantage of metal oxide-based RRAM is the potential for high memory cell density, HfO_2_ based systems provide multilevel cell storage capabilities (Qi et al., 2018; Milo et al., 2019). Achieving stable and repeatable resistive switching behavior is crucial for reliable memory operation. Uniformity of switching characteristics across large arrays of memory cells is also important for commercial viability (Guan et al., 2012). Table 6 presents the comparative study of different properties of graphene and metal oxide-based RRAM devices.

**Table 6:** Comparative analysis of graphene-based RRAM and metal-based RRAM devices.

**Device name**	**Reference**	**Bottom electrode**	**Top electrode**	**Active layer material**	**Switching speed**	**Endurance**	**Retention time**
Graphene Oxide based RRAM	Wang et al. (2012b)	Pt	Al	GO	10^4^	10^2^	10^3^
	Pradhan et al. (2016)	Al	Al	GO	10^2^	10^2^	10^3^
	Wang et al. (2012a)	ITO	Al	GO	10^2^	10^2^	10^4^
Metal oxide-based RRAM	Park et al. (2012)	Graphene	Pt	SiOx	10^2^	80	10^3^
	Yao et al. (2012)	ITO	Graphene	SiO_*x*_	10^5^	10^2^	10^5^
	Tsigkourakos et al. (2017)	TiN/Ti	Au	TiO_2−*x*_	-	>50 cycles	10^5^
	Wu et al. (2018)	Pd	TiN	HfO_*x*_ /Ag/NPs	-	-	10^4^
	Chen et al. (2017)	Al	Al	HfO_*x*_	10^4^	-	-

## 7. RRAM for multi-level cell storage

Multilevel cell storage in RRAM helps to increase the storage density of the memory cell without reducing its size of. In the normal method, the cell size needs to be reduced to increase the density, which requires complex patterning techniques. In the case of multilevel cell storage, the number of bits stored per cell can be increased to n (any integer above 2), increasing the density to n times with 2^*n*^ number of available states in the cell. Among the different memory devices such as Spin Transfer Torque RAM (STTRAM) and phase change memory. RRAM shows excellent scalability beyond the 10 nm technology node. The resistive switching mechanism in RRAM helps to attain different intermediate levels by varying the programming current. The size of the conducting filament in an RRAM device depends directly on the applied current. Thus, by adjusting the value of the current, different resistance states can be attained in the system.

**Figure 17:**
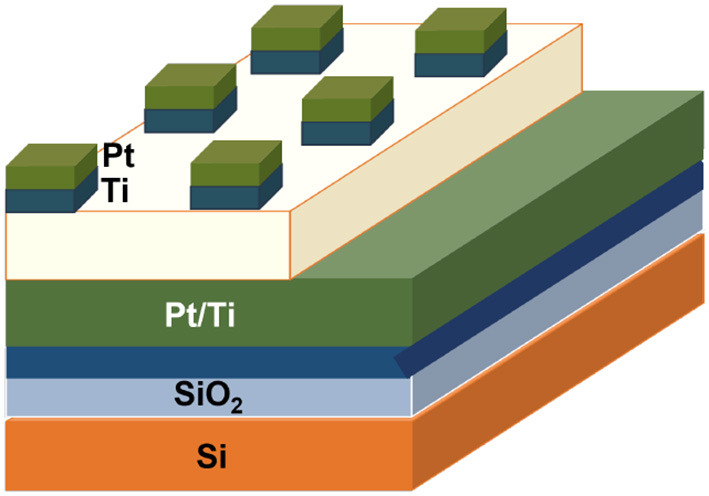
Schematic structure of the Ti/ZrO_2_/Pt RRAM device (Lei et al., 2014).

The multilevel cell storage can be attained via different methods such as (i) varying compliance current, (ii) adjusting reset voltage, and (iii) changing the pulse width of program/erase operation (Prakash and Hwang, 2016). The most common method among these is the controlling of compliance current to obtain multi-level cell storage. The effect of compliance current on the switching mechanism of the Ti/ZrO_2_/Pt is studied by Lei et al. (2014), and the device structure is as shown in Figure 17. In the Ti/ZrO_2_/Pt device architecture, the multilevel cell storage is achieved by controlling the magnitude of the compliance current. The observed multilevel cell storage is explained using the voltage divider rule in a series circuit model. By varying the compliance current, the number of traps in the device is controlled; hence, the conductance is varied. A low voltage four-level cell storage is attained in Ta_2_O_5_/TiO_2_ system by controlling the *R*_*L*_ and *R*_*S*_ state of the device (Terai et al., 2010). They found that multilevel cell storage can be achieved by varying the reset voltage as well. In this study, Ru et al. is used as the top and bottom electrode, and the combination of Ta_2_O_5_/TiO_2_ is used as the middle layer. This device achieved a 2-bit/cell storage by multi R_*H*_ level operation. In another study of the HfO_2_-based RRAM system, the multilevel cell storage is achieved by controlling either I_*set*_ or V_*stop*_ (Lee et al., 2008a).

In order to obtain the stable states in the multilevel cell storage system, it is important to distinguish the reference states from one another. The factors affecting the stability of resistance states are cycle-to-cycle variability, device-to-device variability, operation temperature, random telegraph noise, and interstate switching variability. The study of the retention characteristics and endurance of the device will help to understand the reliability of the multiple resistance levels. It is observed that the retention time for the low resistance state highly depends on the operating current of the device (Ninomiya et al., 2013). With the incorporation of graphene, it is expected to obtain multiple states in the RRAM system. The property of this multi-level cell storage will enable the graphene-based systems to act as a synapse for neuromorphic computing and many other applications.

## 8. Commercially available RRAM models and its fabrication

For several years, researchers have demonstrated the potential of memristive devices in laboratory experiments. As a result, there have been successful demonstrations of these devices in commercial applications, with RRAM devices being particularly noteworthy in solid-state drives (SSDs) and Internet of Things (IoT) devices. Li et al. (2017) proposed a memory-centric computing approach based on RRAM that leverages on-chip non-volatile memories to perform local information processing in a highly energy-efficient manner. Three in-memory operation schemes using 3D RRAM has been developed and experimented to ensure their effectiveness and reliability, allowing for enhanced local information processing that is highly efficient and optimized for memory-centric computing systems. Wang et al. (2018) demonstrated the integration of 1-transistor/1-resistor (1T1R) memory cells using monolayer MoS_2_ transistors and few-layer hBN RRAMs, creating a two-level stacked 3D monolithic structure. The fabrication process was conducted at temperatures below 150 °C. It is observed that this configuration exhibits forming-free (at < 1V) gradual set and reset, where the filament formation process in RRAM is not required for achieving the resistance states and which is particularly advantageous for linear weight updating in neuromorphic computing. However, some renowned company has developed various kind of RRAM devices. Adesto Technologies has recently launched a new chip family called Moneta, which utilizes CBRAM (Conductive Bridging Random Access Memory) technology. The Moneta family offers ultra-low power memory solutions that are designed to significantly reduce the overall energy consumption of connected devices. The chips demonstrate read and write operations at 50-100 times lower power compared to competitive solutions. The company has already begun shipping samples of the Moneta family in four different densities, including 32 Kbit, 64 Kbit, 128 Kbit, and 256 Kbit. Fujitsu recently developed RRAM product which offers 1.5 times higher memory density compared to the existing 8 Mbit RRAM. Other renowned foundries such as Intel, Panasonic, and Samsung have been developing RRAM technology. These companies have been investing heavily in RRAM research and development to improve the performance, reliability, and scalability of this promising memory technology.

## 9. Graphene-based RRAM applications

The researchers are investigating using graphene or graphene oxide (GO) as electrodes or switching material of RRAM targeting in-memory computing for neuromorphic behavior (Izam et al., 2016; Liu et al., 2018; Yan et al., 2018; Abunahla et al., 2020a). The control of resistance for multiple states by memorizing the previous state enables to mimic of biological synapses in the human brain neural network (Sparvoli and Marma, 2018; Xu et al., 2019b; Schranghamer et al., 2020; Kireev et al., 2022). With the large development in memristive materials, an excessive amount of work is being conducted in 2D materials-based memristors for neuromorphic computing (Abunahla et al., 2020a,b; Alimkhanuly et al., 2021). The graphene crossbar variability can be used to build a unique physical unclonable function (PUF), which can be used for various applications. Table 7 presents the review on graphene/GO RRAM for neuromorphic computing.

**Table 7:** A review on graphene RRAM for neuromorphic computing.

**Sl no**.	**Reference**	**Graphene application**	**No. of conductance states**	**Target application**
1	Abunahla et al. (2020a,b)	Au/ partially redued graphene oxide (prGO)/Au	7	ANN of size 5 × 4 and 4 × 4
2	Alimkhanuly et al. (2021)	electrode of 3D vertical RRAM	64	XNOR
3	Sparvoli and Marma (2018)	RRAM fabrication with doped graphene oxide with silver	2	RRAM bridge synapse
4	Schranghamer et al. (2020)	Graphene field effect transistor	16	RRAM synapse
5	Xu et al. (2019b)	Al_2_O_3_/graphene quantum dots/Al_2_O_3_	2	Synapse
6	Kireev et al. (2022)	Bilayer Graphene-based Artificial Synaptic Transistors (BLAST)	100	Synapse transistor

### 9.1. Memory

The characteristic features of RRAM such as simple structure, non-volatile, scalability, low power, and fast operation speed makes it a prominent place for future memory devices. In comparison with other materials, the 2D materials-based RRAM devices offer better transparency and flexibility. The incorporation of graphene will provide more feasible and effective methods to increase the capacity of storage devices. The SET current/voltage, I_*set*_/ V_*set*_, RESET current/voltage, I_*reset*_/ V_*reset*_, resistance ratio R_*OFF*_/ R_*ON*_, programming speed, power, and retention time are the parameters for the evaluation of memory devices. Table 8 shows the list of RRAM architecture in the literature with the evaluation parameters.

**Table 8:** Different RRAM architectures.

**RRAM structure**	**I_*set*_/ V_*set*_**	**I_*reset*_/ V_*reset*_**	**R_*OFF*_/R_*ON*_ Ratio**	**SET/R ESET speed**	**Power**
MLG/Dy_2_O_3_/ITO (Zhao et al., 2014a)	1 μA/0.4V	20 μA /0.2V	>10^5^	60 ns	4.4 μW
Unipolar					
ITO/Al_2_O_3_/ Graphene (Dugu et al., 2018)	2.1 μ*A*/0.8V	1.55 *mA*/-0.65V	~3.5 × 10^3^	NA	~1*mW*
Bipolar					
Al_2_O_3_/GQD/Al_2_O_3_ (Xu et al., 2019b)	< 5*nA*/1.2V	< 5*nA*/-1.2V	NA	NA	NA
ITO/GO+0.1 % Ag/Al (Sparvoli and Marma, 2018)	< 4.78*mA*/0.8V	2 *pA*/0.25V	7.5 × 10^8^	10μ*s*	NA
Unipolar					
G/SiO_*x*_/ITO (Yao et al., 2012)	2 μ*A*/4.26V	2 *mA*/10V	10^4^	50*ns*	20 *mW*
Unipolar					
Au/prGO/Au (Abunahla et al., 2020a,b)	25 *mA*/3V	10 *mA*/-6.5V	10	10*s*	NA
Unipolar					
TiN/HfO_*x*_/ Graphene (Alimkhanuly et al., 2021)	< 1μ A/1.27V	< 10μA/ -1.37V	>10*X*	500 ns	NA
bipolar					

MLG, multi-layer graphene; ITO, indium tinoOxide; GQD, graphene quantum dots; NA, not available.

Zhao et al. (2014a) experimentally demonstrated that the graphene electrode layer provides high built-in series resistance to exhibit good device-to-device uniformity. This exhibits narrow resistance/voltage variations in both ON and OFF states. The switching characteristics of ITO/Al_2_O_3_/Graphene RRAM is compared with ITO/Al_2_O_3_/Pt RRAM devices in Dugu et al. (2018). The results in Dugu et al. (2018) show that graphene shows a low SET/RESET current/voltage in comparison with conventional RRAM electrodes such as Pt. A perceptron model is experimentally in Sparvoli and Marma (2018).

Lu et al. (2022) have developed a two-terminal memristor synapse based on a silicon-argon composite film. In the case of the biological synapse, the weight is varied by the release of neurotransmitters from the preneuron induced by spikes. Thus, similar to that, this memristive synapse varies its conductance by the migration of the ions upon an external electrical signal or stimuli.

### 9.2. Neural networks

**Figure 18:**
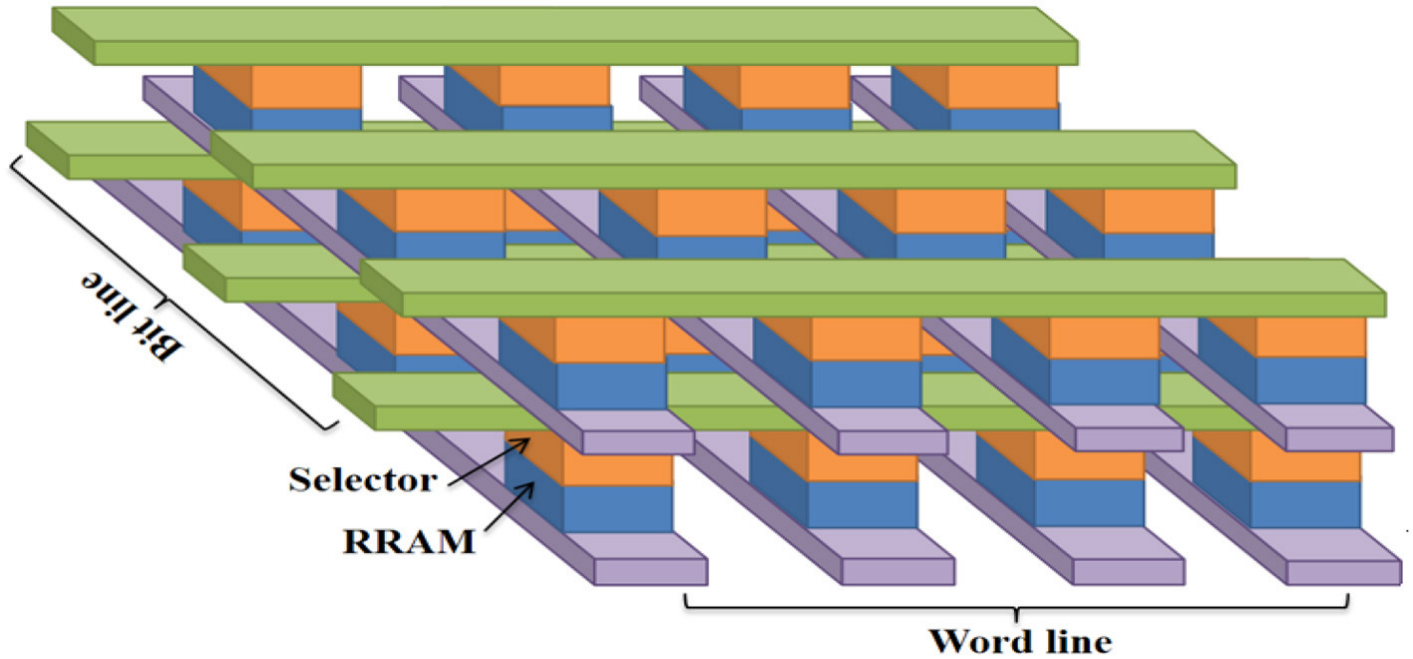
3D RRAM crossbar array.

The RRAM crossbar in-memory computing is considered to be a potential solution for implementing power-efficient neural network architectures (Li et al., 2018; Mehonic et al., 2020). The analog/digital feature of RRAM, with the ability to memorize, can be used to build artificial neural networks for neuromorphic computation (Mehonic et al., 2020). Figure 18 shows crossbar architecture using RRAM devices for realizing the neuromorphic computations. The weights of neural computations are programmed onto the RRAM devices during the write mode. Only a few studies have been reported in the literature using graphene/GO-based RRAM for neuromorphic computing (Abunahla et al., 2020a,b; Alimkhanuly et al., 2021). Both 2D and 3D crossbar architecture with RRAM have been discussed in the literature for neuromorphic computing. HebaAbunahla et al. presented a novel planar analog memristor crossbar with partially reduced graphene oxide (prGO) thin film (Abunahla et al., 2020a,b). In Abunahla et al. (2020a,b), the crossbar array has been fabricated and tested using the Iris dataset with an accuracy of 96.67%. 5 × 4 and 4 × 4 crossbar arrays have been fabricated, which is then used to classify the iris flower based on its petal and sepal length and width into different classes.

Alimkhanuly et al. (2021) demonstrated a 3D vertical RRAM (VRRAM) by replacing the metal-based interconnects with graphene due to the remarkable electronic and thermal conductivities. In Alimkhanuly et al. (2021), the authors fabricated a 416 × 224 × 8 size 3D array system. The recognition performance of the fabricated 3D graphene RRAM (Gr-RRAM) has been tested for the MNIST dataset. The network size is 400 input, 200 hidden, and 10 output neurons. The performance accuracy of Gr-RRAM is compared with platinum RRAM (Pt-RRAM), and the results show that the overall accuracy levels degrade for Pt-RRAM due to high read inaccuracy.

### 9.3. Logic gates

The logic computing is yet another application of memristor crossbar structure. The XOR operation-focused 3D VRRAM array architecture is demonstrated in Alimkhanuly et al. (2021). The XOR architecture using graphene-based VRRAM arrays have the potential of a highly stackable nature for parallel processing of multiple layers (Alimkhanuly et al., 2021). An XNOR logic-inspired architecture is designed to integrate 1-bit ternary precision synaptic weights into graphene-based VRRAM is presented in Alimkhanuly et al. (2021). However, robustness to device variability by using graphene-based RRAM in logic computing is not yet investigated in the literature and still remains an open problem.

### 9.4. Cryptography

The memristor crossbar arrays is also applied for cryptography applications (Cai et al., 2022; Yu et al., 2023). An in-memory hyperdimensional encryption using a memristor crossbar array is presented in Cai et al. (2022). The robustness of binary hypervectors against memristor crossbar non-ideality helps to control the impact of noise generated by the memristor crossbar for encryption. A 4D memristive hopfield neural network (MHNN) is proposed in Yu et al. (2023) for image encryption applications. The majority of memory-based cryptographic techniques for hardware security are based on physical unclonable functions (PUFs) (James, 2019). A large number of memory-crossbar-based PUFs have been proposed in the literature, for example, metal-oxide memristor-based or RRAM (Rose and Meade, 2015; Yansong et al., 2015; Uddin et al., 2017; Khan et al., 2021; Kim et al., 2021) etc. The PUF methods use variations in device parameters such as resistance state, switching time, and threshold voltages. These unpredictable probabilistic characteristics of memristor crossbars form the basis for PUF applications. The variations in device parameters and process variations affect the current flow through the device. Any temporal or spatial variations affect all aspects of resistive switching. The variation in PUF characteristics with the properties of graphene has not been explored yet in the literature.

The stochasticity in graphene-RRAM device response has not been extensively studied in the existing literature. The repeatability of fabricated Gr-RRAM devices are experimentally evaluated in the literature. The SET voltage varies for cycle-cycle variations for Gr-RRAM was found to be 6.4% in Alimkhanuly et al. (2021). As discussed in Kim et al. (2021), the SET voltage variations in Gr-RRAM crossbar array can also be used for PUF generation in cryptographic applications. The other device variations such as resistance state, switching time, and threshold voltages have not been considered for analysis with device-to-device and cycle-to-cycle variations. The stochasticity in graphene-RRAM variation for cryptography or PUF characteristics has not been explored yet in the literature and is an open problem.

## 10. CMOS compatibility

CMOS technology faces various unwanted problems due to the scaling of device attributes. The semiconductor industry is planning to replace the silicon material with graphene material. Since graphene is a conducting material and no energy band gap is present in it, it is very difficult to use graphene for digital device applications due to high-off state leakage and non-saturating drive currents. However, graphene-based devices are more acceptable for low-noise amplifiers and radio-frequency (RF) in analog device applications (Banerjee et al., 2010). Rodriguez et al. (2012) compared the RF behavior between graphene-based field effect transistor (GFET) and Si-based metal oxide field effect transistor (MOSFET). It is observed that the GFET device is more acceptable for the narrow range of drain voltage and drain current compared to Si-MOSFET. Cisneros-Fernández et al. (2019) proposed frequency domain multiplexing of liquid-gate GFET sensor for micro electrocorticogram (ECoG) recording purpose. The proposed work also allows hybrid integration.

Nowadays, graphene with Si CMOS circuits can also be constructed together for making heterogeneous devices. The demonstration of graphene and Si CMOS hybrid circuits has reduced barriers to entry of graphene in electronics. Huang et al. (2014) constructed a low-temperature hybrid integrated circuit where graphene devices and Si-CMOS circuits integrated together. Gilardi et al. (2019) designed relaxation oscillators using a GFET, Si CMOS D latch, and timing RC circuit. It is observed that the introduction of graphene material in the Si-CMOS logic circuit has improved the circuit complexity and also added other device functionality. One of the truly unique electronic properties of graphene not exhibited by conventional semiconductors is ambipolarity. The ambipolarity of graphene helps to simplify the circuit and provide additional functionality. Graphene's ambipolarity eliminates the need for separate electron and hole transistors, reducing the overall transistor count and circuit complexity (Jabeur et al., 2010). The integration of graphene into Si CMOS logic circuits could offer a feasible approach for both simplification and enhanced functionality. Zhang et al. (2014b) proposed CMOS-compatible all-metal-nitride RRAM based on aluminum nitride (AlN). It is observed that the proposed device provides a lower operation current of 100 A, retention time 3x10^5^, and endurance value of 10^5^ Hz. AlN has high thermal stability, allowing it to withstand the elevated temperatures used in CMOS processes. This makes it possible to integrate AlN-based RRAM fabrication steps into standard CMOS processes without causing significant damage to the underlying circuitry (Jackson et al., 2013). AlN can be deposited using various techniques that are already employed in CMOS manufacturing, such as PVD and CVD (Perez-Campos et al., 2015). PVD and CVD methods allow for conformal deposition of thin AlN films over complex three-dimensional structures, including the intricate features found in modern CMOS circuits (Cansizoglu et al., 2015). This conformal deposition capability is crucial for integrating RRAM cells within the existing CMOS architecture. The temperature requirements and chemical interactions during AlN deposition are generally more manageable compared to some graphene synthesis methods. Graphene-based RRAM, on the other hand, could face more integration challenges due to the specialized processes required for graphene synthesis and transfer. Graphene synthesis and transfer techniques involve high-temperature processes and chemical treatments that could affect the performance of the graphene itself (Choi et al., 2022). Achieving high-quality, defect-free graphene layers on a large scale while maintaining CMOS compatibility remains a significant hurdle (Moon and Gaskill, 2011). Yeh and Wong (2015) proposed a cost-competitive One-Transistor-N-RRAM (1TNR) array architecture for advanced CMOS technology where one committed transistor controls the access of one RRAM. It is observed that the 1TNR array architecture provides less leakage current than the cross-point array. Therefore, there is the possibility that graphene-based RRAM memory devices can be considered in CMOS technology soon.

## 11. Challenges and future scope

Due to its unique and interesting features, graphene has surpassed all other nanomaterials in terms of its use in electronic devices. Additionally, it was shown that graphene's greater mobility, less light absorption, and excellent mechanical qualities enhance the functionality of transparent flexible electronic devices. The difficulty is that the cost of manufacturing graphene will increase the overall price of the device. The transfer of graphene from one substrate to another without causing any damage is a tedious process, which requires the need of sophisticated instruments. Efficient methods need to be implemented to overcome these drawbacks.

The past several years have seen a substantial increase in research into new memory technologies, and numerous prototype RRAM products have been created to show the potential for high-speed and low-power applications. The CMOS compatibility and ability to fabricate in smaller dimensions make the RRAM a suitable candidate for device applications. A high endurance is reported in graphene-based RRAM devices. To date, in a single RRAM device, no technology has reported fast switching, low power, and stable operation simultaneously. In a graphene-based RRAM device, the properties need to be enhanced for better performance of the device.

## 12. Conclusion

This review article offers an insightful look into the topic of developing graphene-based RRAM devices in terms of neural computing by giving a concise overview of the development of memory architecture, the current trends, and the constraints. The importance of graphene based RRAM, as well as its structure, operation, and classification, have all been highlighted in a thorough discussion. The methodology and a detailed investigation on the MLC capabilities of RRAM have been presented. It is proposed that the graphene-based RRAM can be used for multilevel cell storage. This modified memory device, with 2D material can be used as a synapse. Along with this, the implementation of graphene based RRAM for various important applications such as hardware security and neuromorphic computing have been highlighted.

## Author contributions

RTR, RD, and CR: Conducted literature review, prepared a part of the draft copy of the manuscript, and revision of the manuscript. AJ: Contributed to the theoretical framework development, provided critical insights during data analysis and interpretation, manuscript correction and writing, funding acquisition and supervision.
